# Non-homogeneous dynamic Bayesian networks with edge-wise sequentially coupled parameters

**DOI:** 10.1093/bioinformatics/btz690

**Published:** 2019-09-05

**Authors:** Mahdi Shafiee Kamalabad, Marco Grzegorczyk

**Affiliations:** Bernoulli Institute, Department of Mathematics, Faculty of Science and Engineering, Groningen University, Groningen 9747 AG, The Netherlands

## Abstract

**Motivation:**

Non-homogeneous dynamic Bayesian networks (NH-DBNs) are a popular tool for learning networks with time-varying interaction parameters. A multiple changepoint process is used to divide the data into disjoint segments and the network interaction parameters are assumed to be segment-specific. The objective is to infer the network structure along with the segmentation and the segment-specific parameters from the data. The conventional (uncoupled) NH-DBNs do not allow for information exchange among segments, and the interaction parameters have to be learned separately for each segment. More advanced coupled NH-DBN models allow the interaction parameters to vary but enforce them to stay similar over time. As the enforced similarity of the network parameters can have counter-productive effects, we propose a new consensus NH-DBN model that combines features of the uncoupled and the coupled NH-DBN. The new model infers for each individual edge whether its interaction parameter stays similar over time (and should be coupled) or if it changes from segment to segment (and should stay uncoupled).

**Results:**

Our new model yields higher network reconstruction accuracies than state-of-the-art models for synthetic and yeast network data. For gene expression data from *A.thaliana* our new model infers a plausible network topology and yields hypotheses about the light-dependencies of the gene interactions.

**Availability and implementation:**

Data are available from earlier publications. Matlab code is available at *Bioinformatics* online.

**Supplementary information:**

Supplementary data are available at *Bioinformatics* online.

## 1 Introduction

One of the key objectives of computational systems biology is to learn the structure of protein activation pathways and gene regulatory networks. With the work of Friedman *et al.* (2000), dynamic Bayesian networks (DBNs) have become a popular tool for learning networks from data. However, DBNs are homogeneous linear models that in some applications cannot satisfactorily approximate the complexity of real gene regulatory interaction relationships. Hence, there can be gains from more flexible network reconstruction models. For example, in cellular networks the strengths of the regulatory interactions can depend on unobserved cellular conditions that are not constant in time, so that the application of a homogeneous model (DBN) would be suboptimal. For modelling time-varying regulatory networks many non-homogeneous DBNs (NH-DBNs) have been proposed in the literature. Those NH-DBN models can be divided into two conceptual groups: (i) NH-DBNs that only allow the network parameters to vary in time (see references below) and (ii) NH-DBNs that allow even the network structure to be time-dependent (see, e.g. Husmeier *et al.*, 2010; Lèbre *et al.*, 2010; Robinson and Hartemink, 2010). The latter group (ii) offers great model flexibility, but faces a practical and a conceptual problem. The practical problem is potential model over-flexibility. Time series in systems biology are typically rather short and NH-DBNs divide them into even shorter segments. Learning different network structures for short segments that contain a few data points only is a challenging task and likely to lead to inflated inference uncertainty. The conceptual problem is related to the very premise of a flexible network structure. This assumption is surely reasonable for some scenarios, like morphogenesis. See, for example, the application to morphogenesis and muscle growth in *D.melanogaster* in Robinson and Hartemink (2010), where the gene expression time series cover the embryonic, larval, pupal and adult life phase of the fruit fly. Obviously, a gene regulatory network in an embryonic fruit fly can change during growth to maturity and eventually have another structure with different gene interactions in an adult fruit fly. However, for cellular processes on a short time scale, it is questionable whether the network structure can vary over time. By convention, an edge from gene *Z_i_* to gene *Z_j_* in a gene regulatory network indicates that gene *Z_i_* codes for a transcription factor that can bind to the promoter of gene *Z_j_*, so as to initiate its transcription. This biological ability to bind is unlikely to change within a short time period. In a short period of time, only the extent of binding is likely to be influenced by changing external factors (e.g. cellular conditions), so that only the strength of the regulatory effect can vary over time.

We therefore focus on NH-DBNs of group (i), where the network structure is assumed to be time-invariant. In particular, this assumption is more realistic for our two real-world applications to *S.cerevisiae* (yeast) and to *A.thaliana* (plant) gene expression data. In the metabolism-related gene regulatory network in yeast (Section 5.2) the strengths of the regulatory interactions depend on the medium, in which yeast is cultured (galactose and glucose). In the circadian clock network in Arabidopsis (Section 5.3) the strengths of the gene regulatory interactions depend on the artificially generated dark: light cycles, to which the plants were earlier exposed.

The NH-DBN models infer the data segmentation, the joint network structure and the segment-specific interaction parameters altogether from the data. As already pointed out above, in typical applications, those NH-DBNs divide a short time series into even shorter segments. Learning the network parameters for each segment separately can then also lead to inflated inference uncertainty. Therefore models that allow for gradual adaptions of the network interaction parameters have been proposed. The TESLA method (Ahmed and Xing, 2009; Kolar *et al.*, 2010) makes use of L1-regularized regression models (‘LASSO’) for the network parameter inference, and it employs a second L1 regularization term to penalize dissimilarities between the network parameters of neighbouring segments. Inference is based on a penalized maximum likelihood approach, and the regularization parameters can be optimized by the Bayesian information criterion (BIC) or cross-validation. TESLA even allows the network structure to be time-dependent. But as changing network structures yield large L1 penalties, the network structure is encouraged to stay similar.

The NH-DBN model from Grzegorczyk and Husmeier (2012) uses Bayesian hierarchical regression to sequentially couple the parameters. The resulting coupled NH-DBN can be seen as a Bayesian counterpart of TESLA. In the simulation study by Aderhold *et al.* (2014) the Bayesian NH-DBN yielded better network predictions than TESLA.

It has also been shown that parameter coupling leads to significantly improved network predictions when the segment-specific parameters are similar (Grzegorczyk and Husmeier, 2012). However, our empirical results in Section 5.1 show that coupling can be counter-productive when the segment-specific parameters are dissimilar.

The disadvantage of all proposed coupling schemes is that they have been designed such that they can only couple *all* interaction parameters simultaneously. If a node *Z_k_* is regulated by two nodes, $Z_{i}\to Z_{k}\leftarrow Z_{j}$, then the parameters for both edges are coupled with the same coupling strength. But the effect of *Z_i_* on *Z_k_* could stay similar, while the regulatory effect of *Z_j_* on *Z_k_* could be subject to significant temporal changes.

Given the complexity of the interactions in gene regulatory networks, it might thus be useful to add more flexibility to the models. In this paper we therefore propose a new consensus model with an edge-wise coupling (EWC) scheme. Unlike the coupled NH-DBN, the new EWC NH-DBN does not enforce coupling. Instead it follows the Bayesian paradigm: ‘*Let the data speak.*’ and infers for each individual edge (edge-wise) if the corresponding interaction parameter should be coupled or not.

The EWC NH-DBN has the uncoupled and the coupled NH-DBN as limiting cases and it can infer an appropriate trade-off between them. In addition, the EWC NH-DBN can also shed more light onto the robustness of the individual regulatory interactions. Instead of enforcing a priori that either all edges are coupled or that all edges are uncoupled, it infers for each individual edge whether it should be coupled or better stay uncoupled. From a biological perspective, one can conclude that an uncoupled edge is sensitive to external factors, as the interaction parameter (i.e. the strength of the regulatory effect) varies over time. On the other hand, the interaction parameter of a coupled edge stays (rather) stable, so that the strength of the regulatory effect is not (or only minimally) influenced by external factors. For the circadian clock network in Arabidopsis this feature of the EWC NH-DBN can lead to important new insights. One of the objectives of computational plant biology is to derive a faithful description of the circadian clock network in terms of coupled differential equations (DEs); see, e.g. the work by Pokhilko *et al.* (2013). The diurnal rhythm of the circadian clock network is caused by the actual (or entrained) daily dark:light cycles, as some of the gene interactions are intensified or alleviated by the presence (or expectation) of light. The DE models therefore typically contain an additional light variable that has an effect on some of the regulatory interactions. For an overview of different network hypotheses from the plant biology literature, we refer to Figure 12 in Aderhold *et al.* (2014). In this overview figure a ‘sun symbol’ is used to indicate the effects of light within the different circadian clock network hypotheses. Because of the computational costs, the space of all possible network structures cannot be systematically searched with DEs. In typical studies, based on prior knowledge a few novel network structure hypotheses are proposed and then compared with earlier published network hypotheses. As the computational costs allow only a few new hypotheses to be included, the new network structures must be carefully selected and it must also be carefully decided which gene interactions are supposed to be affected by the presence of light (see, e.g. Pokhilko *et al.*, 2013).

Unlike DEs, NH-DBNs can be used to learn the complete network structure from scratch, and thus help generating new hypotheses about it. Unlike all earlier proposed NH-DBNs, the new EWC NH-DBN employs an edge-wise coupling concept and can distinguish between regulatory effects that are stable (coupled) and regulatory effects that are unstable (uncoupled). In the circadian clock, the instability of an edge suggests that the corresponding gene interaction is likely to be light-dependent. This knowledge about the (in-)stability of the regulatory interactions is therefore useful information for subsequent DE modelling approaches. It can be used as prior knowledge when deciding about the light dependency of the edges of a newly proposed DE-based network hypothesis.

In our recent work (Shafiee Kamalabad *et al.*, 2019), we have proposed a partially non-homogeneous DBN for learning networks from a collection of datasets that have been measured under different experimental conditions. The model assumes the data segmentation to be known (one segment per condition), and then treats the segments as interchangeable units. The EWC NH-DBN focuses on network time series with unknown segmentations. Unlike the earlier model, the EWC NH-DBN infers the segmentation from the data, and then uses the temporal order of the segments. Given the order, coupling can be applied sequentially, so that every segment receives information from the preceding one. This allows for gradual/smooth temporal adaptions of the parameters. Another conceptual difference is that the earlier model is partially non-homogeneous, while the EWC NH-DBN is strictly non-homogeneous with an edge-wise sequential information-coupling scheme for the interaction parameters.

We now briefly return to the work by Friedman *et al.* (2000), in which DBNs were proposed for learning gene networks. Since then DBNs have become a popular tool for network learning, although they are based on two simplifying assumptions, namely that the interactions are *homogeneous* and *linear*. For gene regulatory interactions, both assumptions can be too restrictive. Above we have discussed model extensions that relax the homogeneity assumption, but none of those methods makes an attempt to relax the linearity assumption. In a complementary line of research, authors have proposed methods that keep the homogeneity assumption but relax the linearity assumption, so that homogeneous non-linear gene interactions can be inferred. For example, Oates and Mukherjee (2012) have added quadratic and interaction terms to the design matrices of linear models. Other non-linear methods make use of Gaussian process regression (Äijö and Lähdesmäki, 2009), non-parametric additive models (Henderson and Michailidis, 2014) or faithful descriptions of the gene interaction kinetics in form of differential equations (Aderhold *et al.*, 2017; Oates *et al.*, 2014). We briefly describe these methods in Section 2.7 and we also compare the performance of the EWC NH-DBN with them. We illustrate the conceptual difference between non-homogeneous linear and homogeneous non-linear models in Supplementary Material Part I. To the best of our knowledge, no non-homogeneous non-linear method has been proposed yet.

## 2 Materials and methods

### 2.1 The new edge-wise coupling (EWC) scheme

Consider a Bayesian piece-wise linear regression model with *Y* being the response and $\pi =\{X_{1},\ldots ,X_{k}\}$ being a set of covariates. We assume that the data points have a temporal order and can be divided into disjoint segments h∈{1,…,H}, where each segment *h* has specific regression coefficients, $\beta_{h}={\left(\beta_{h,0},\ldots ,\beta_{h,k}\right)}^{T}$. Let $y_{h}$ be the vector of the response values and $X_{h}$ be the design matrix for segment *h*, where each $X_{h}$ includes a first column of 1’s for the intercept. For each segment *h* we use a Gaussian likelihood:

(1) $$y_{h}|(\beta_{h},\sigma^{2})∼N(X_{h}\beta_{h},\sigma^{2}I)\;\;\;\;\;(h=1,\ldots ,H)$$

where I denotes the identity matrix, and $\sigma^{2}$ is the noise variance parameter, which is shared among segments. We impose an inverse Gamma prior on $\sigma^{2},\;\sigma^{-2}∼\mathrm{GAM}(a_{\sigma },b_{\sigma })$, and a Gaussian prior on $\beta_{1}$:

(2) $$\beta_{1}|(\sigma^{2},\lambda_{u})∼N(0,\sigma^{2}\lambda_{u}I)$$

where $0:={\left(0,\ldots ,0\right)}^{T}$. Onto the ‘*signal-to-noise ratio parameter for uncoupled regression coefficients*’, *λ_u_*, we also impose an inverse Gamma distribution, $\lambda_{u}^{-1}∼\mathrm{GAM}(a_{u},b_{u})$. Re-employing $\sigma^{2}$ in Equation (2) yields a fully conjugate prior in both $\beta_{1}$ and $\sigma^{2}$, so that the marginal likelihood $p(y_{1}|\lambda_{u})$ can be computed (see, e.g. Gelman *et al.*, 2004). The posterior distribution of $\beta_{1}$ is:

(3) $$\beta_{1}|(y_{1},\sigma^{2},\lambda_{u})∼N(\tilde{\beta_{1}},\sigma^{2}C_{1})$$

where $C_{1}={\left({\left[\lambda_{u}I\right]}^{-1}+X_{1}^{T}X_{1}\right)}^{-1}$, and ${\tilde{\beta }}_{1}=C_{1}X_{1}^{T}y_{1}$ is the posterior expectation of $\beta_{1}$. If we use the same Gaussian prior for all segments

(4) $$\beta_{h}|(\sigma^{2},\lambda_{u})∼N(0,\sigma^{2}\lambda_{u}I)\;\;\;\;(h=1,\ldots ,H)$$

we obtain an uncoupled model. The only information exchange among segments is then be w.r.t. the shared parameters $\sigma^{2}$ and *λ_u_*.

If we use the posterior expectation ${\tilde{\beta }}_{h}$ as prior expectation for $\beta_{h+1}$:

(5) $$\beta_{h+1}|(\sigma^{2},\lambda_{c},{\tilde{\beta }}_{h})∼N({\tilde{\beta }}_{h},\sigma^{2}\lambda_{c}I)\;\;\;\;(h=2,\ldots ,H)$$

we obtain a sequentially coupled model. The parameter *λ_c_* is then a ‘*coupling strength parameter*’, for which we could assume an inverse Gamma distribution, $\lambda_{c}^{-1}∼\mathrm{GAM}(a_{c},b_{c})$. ‘*Coupling*’ here means that $\beta_{h+1}$ is coupled to the posterior expectation ${\tilde{\beta }}_{h}$ of $\beta_{h}$. Low values *λ_c_* yield peaked priors in Equation (5), so that the vectors $\beta_{h}$ and $\beta_{h+1}$ are enforced to be similar (=coupled). Dissimilar regression coefficients can only be obtained for large values of *λ_c_*, i.e. for vague priors in Equation (5). The shortcoming is that there is no distinction between the individual regression coefficients: they are all coupled with the same coupling strength (via the parameter *λ_c_*).

In this paper, we propose a new model that infers a consensus between Equations (4 and 5). The new NH-DBN infers from the data which regression coefficients stay similar over time (and should be coupled) and which regression coefficients change significantly over time (and should stay uncoupled). In each segment the uncoupled regression coefficients will be re-initialized non-informatively with a prior expectation of 0 and the corresponding prior variance will depend on the signal-to-noise ratio parameter *λ_u_* from Equation (4) rather than on the coupling strength parameter *λ_c_* from Equation (5). We refer to the new model as the edge-wise coupled (EWC) NH-DBN.

To distinguish between coupled and uncoupled regression coefficients, we introduce a vector of indicator variables $\delta =(\delta_{0},\ldots ,\delta_{k})$ whose elements are binary variables $\delta_{i}\in \{0,1\}$: *δ*_0_ corresponds to the intercept, and *δ_i_* (i≥1) refers to the *i*th covariate *X_i_*. $\delta_{i}=1$ indicates that the *i*th regression coefficients $\beta_{1,i},\ldots ,\beta_{H,i}$ are coupled, while $\delta_{i}=0$ indicates that they are uncoupled. We introduce the new Gaussian prior:

(6) $$\beta_{h+1}|(\sigma^{2},\lambda_{u},\lambda_{c},{\tilde{\beta }}_{h},\delta )∼N(\delta ⊙{\tilde{\beta }}_{h},\sigma^{2}\cdot \mathrm{diag}\{\lambda_{c}\delta +\lambda_{u}(1-\delta )\})$$

where ⊙ is the Hadamard product (‘elementwise multiplication’), diag{x} denotes a diagonal matrix whose diagonal elements are the elements of the vector x, and $1:={\left(1,\ldots ,1\right)}^{T}$. As the covariance matrix in Equation (6) is a diagonal matrix, each element $\beta_{h+1,i}$ of $\beta_{h+1}$ is independently Gaussian distributed:

(7) $$\beta_{h+1,i}|(\sigma^{2},\lambda_{u},\lambda_{c},{\tilde{\beta }}_{h,i},\delta_{i})=\{\begin{array}{c} N(0,\;\;\;\;\;\;\sigma^{2}\lambda_{u})\;\text{if}\;\delta_{i}=0\\ N({\tilde{\beta }}_{h,i},\;\sigma^{2}\lambda_{c})\;\text{if}\;\delta_{i}=1 \end{array}$$

where ${\tilde{\beta }}_{h,i}$ is the *i*th element of the posterior expectation ${\tilde{\beta }}_{h}$. The new prior yields a consensus between an uncoupled and a coupled model:

- For δ=0, we have $\beta_{h+1}|(\sigma^{2},\lambda_{u},\lambda_{c},{\tilde{\beta }}_{h},\delta )∼N(0,\sigma^{2}\lambda_{u}I)$ for all *h*, so that the EWC NH-DBN is (fully) uncoupled.
- For δ=1, we have $\beta_{h+1}|(\sigma^{2},\lambda_{u},\lambda_{c},{\tilde{\beta }}_{h},\delta )∼N({\tilde{\beta }}_{h},\sigma^{2}\lambda_{c}I)$ for h≥1, so that the EWC NH-DBN is (fully) coupled.
- The EWC NH-DBN infers $\delta =(\delta_{0},\ldots ,\delta_{k})$ from the data, so as to find an appropriate trade-off between the two limiting models.

A priori we assume $\delta_{0},\ldots ,\delta_{k}$ to be independently Bernoulli distributed: $\delta_{i}∼\mathrm{BER}(p)$(i=0,…,k). The parameter *p* could also be assumed to have a Beta hyperprior, p∼BETA(a,b). For our applications the extension, p∼BETA(1,1), did not lead to improvements over *p *=* *0.5.

**Fig. 1.**
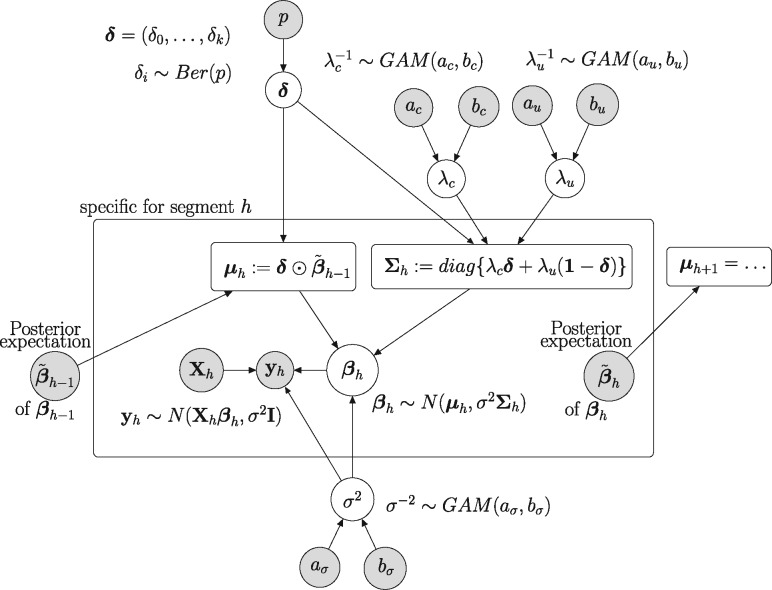
Graphical model representation of the new EWC NH-DBN with edge-wise coupled (EWC) interaction parameters. Variables that have to be inferred are represented by white circles. The data and the fixed hyperparameters are represented by grey circles. The rectangles indicate definitions, which deterministically depend on the parent nodes. All nodes in the inner plate are specific for segment *h*. The posterior expectation ${\tilde{\beta }}_{h-1}$ is treated like a fixed vector when used as input for segment *h *>* *1

Figure 1 shows a graphical model of the EWC NH-DBN, indicating the relationships within and between segments. For the posterior distribution we have:

(8) $$p(\beta_{1},\ldots ,\beta_{H},\sigma^{2},\lambda_{u},\lambda_{c},\delta |y_{1},\ldots y_{H})$$

$$\propto \left(\prod_{h=1}^{H}p(y_{h}|\sigma^{2},\beta_{h})\right)\cdot p(\lambda_{u})\cdot p(\lambda_{c})\cdot p(\sigma^{2})\cdot p(\delta )$$

$$\cdot P(\beta_{1}|\sigma^{2},\lambda_{u})\cdot \left(\prod_{h=2}^{H}P(\beta_{h}|\sigma^{2},\lambda_{u},\lambda_{c},\delta ,{\tilde{\beta }}_{h-1})\right)$$

### 2.2 Gibbs sampling of the model parameters

All free parameters of the EWC NH-DBN (i.e. the white circles in Fig. 1) can be sampled from their full conditional distributions (‘Gibbs sampling’). The derivations of the full conditional distributions (FCDs) are mathematically involved, so that we delegate them to Supplementary Material Part A. Here we just briefly summarize the results. The FCD of $\beta_{1}$ has been provided in Equation (3). For *h *>* *1 we set:

(9) $$\mu_{h}:=\delta ⊙{\tilde{\beta }}_{h-1}\;\;\text{and}\;\;\;\Sigma_{h}:=\mathrm{diag}\{\lambda_{c}\delta +\lambda_{u}(1-\delta )\}$$

so that the priors take the form: $\beta_{h}∼N(\mu_{h},\sigma^{2}\cdot \Sigma_{h})$. We obtain:

(10) $$\mathrm{FCD}(\beta_{h})∼N(C_{h}(\Sigma_{h}^{-1}\mu_{h}+X_{h}^{T}y_{h}),\sigma^{2}C_{h})$$

where $C_{h}={\left(\Sigma_{h}^{-1}+X_{h}^{T}X_{h}\right)}^{-1}$.

The noise variance parameter, $\sigma^{2}$, can be re-sampled via a collapsed (C) Gibbs sampling step, where the regression coefficients, $\beta_{1},\ldots ,\beta_{H}$, have been integrated out:

$$\mathrm{FC}D_{C}(\sigma^{-2})∼\mathrm{GAM}\left(a_{\sigma }+0.5\cdot T,b_{\sigma }+0.5\cdot \Delta^{2}\right)$$

where *T* is the total number of data points from all response vectors, and

$$\Delta^{2}:=\sum_{h=1}^{H}{\left(y_{h}-X_{h}\mu_{h}\right)}^{T}{\left(I+X_{h}\Sigma_{h}X_{h}^{T}\right)}^{-1}(y_{h}-X_{h}\mu_{h})$$

where $\mu_{h}$ and $\Sigma_{h}$ were defined in Equation (9). In Equation (9) we have ${\tilde{\beta }}_{0}:=0$, and ${\tilde{\beta }}_{h}={\left(\Sigma_{h}^{-1}+X_{h}^{T}X_{h}\right)}^{-1}(\Sigma_{h}^{-1}\mu_{h}+X_{h}^{T}y_{h})$ is the posterior expectation of $\beta_{h}$ (h≥1).

For $\lambda_{u}^{2}$ and $\lambda_{c}^{2}$ we obtain the full conditional distributions:

$$\begin{array}{c} F\mathrm{CD}(\lambda_{u}^{-1})∼\mathrm{GAM}\left(a_{u}+\frac{k_{u}}{2},b_{u}+\frac{1}{2}\sigma^{-2}D_{u}^{2}\right)\\ F\mathrm{CD}(\lambda_{c}^{-1})∼\mathrm{GAM}\left(a_{c}+\frac{k_{c}}{2},b_{c}+\frac{1}{2}\sigma^{-2}D_{c}^{2}\right) \end{array}$$

where

$$\begin{array}{c} D_{u}^{2}:=\sum_{i=0}^{k}\beta_{1,i}^{2}+\sum_{h=2}^{H}\;\;\sum_{i:\delta_{i}=0}\beta_{h,i}^{2}\\ D_{c}^{2}:=\sum_{h=2}^{H}\;\;\sum_{i:\delta_{i}=1}{\left(\beta_{h,i}-{\tilde{\beta }}_{h-1,i}\right)}^{2}\\ k_{u}:=(k+1)+(H-1)\cdot \sum_{i=0}^{k}\left(1-\delta_{i}\right)\\ k_{c}:=(H-1)\cdot \sum_{i=0}^{k}\delta_{i} \end{array}$$

so that *k_u_* and *k_c_* are the numbers of uncoupled and coupled regression coefficients with $k_{u}+k_{c}=H\cdot (k+1)$.

For the marginal likelihood, with $\beta_{1},\ldots ,\beta_{H}$ and $\sigma^{2}$ integrated out, we get (Bishop, 2006):

(11) $$\begin{array}{c} p(y_{1},\ldots ,y_{H}|\lambda_{u},\lambda_{c},\delta )\\ =\frac{\Gamma (\frac{T}{2}+a_{\sigma })}{\Gamma (a_{\sigma })}\cdot \frac{\pi^{-T/2}\cdot {\left(2b_{\sigma }\right)}^{a_{\sigma }}\cdot {\left(2b_{\sigma }+\Delta^{2}\right)}^{-(\frac{T}{2}+a_{\sigma })}}{{\left(\prod_{h=1}^{H}\det(I+X_{h}\Sigma_{h}X_{h}^{T})\right)}^{1/2}} \end{array}$$

where $\Delta^{2}$ and $\Sigma_{h}$ (h=1,…,H) were defined above.

For the elements of the vector $\delta =(\delta_{0},\ldots ,\delta_{k})$ we get:

(12) $$\mathrm{FCD}(\delta_{i})∼\mathrm{BER}(\theta_{i})$$

where $\theta_{i}=\frac{p(y_{1},\ldots ,y_{H}|\lambda_{u},\lambda_{c},\delta^{\delta_{i}\leftarrow 1})\cdot p}{\sum_{j=0}^{1}p(y_{1},\ldots ,y_{H}|\lambda_{u},\lambda_{c},\delta^{\delta_{i}\leftarrow j})\cdot p^{j}\cdot {\left(1-p\right)}^{1-j}}$ and $\delta^{\delta_{i}\leftarrow j}$ is the vector δ with *δ_i_* being set to j∈{0,1}.

### 2.3 Learning the covariate set and the data segmentation

In typical applications, the covariate set and the data segmentation are unknown and have to be inferred from the data. Let D denote a time series of equidistant data points, indexed t=1,…,T. Each data point $D_{t}$ contains a response observation *y_t_* and the observations $x_{t,1},\ldots ,x_{t,n}$ of *n* potential covariates. We assume all covariate sets $\pi \subset \{X_{1},\ldots ,X_{n}\}$ to be equally likely a priori, subject to the ‘fan-in constraint’: |π|≤3.

As prior on the number of segments *H* we take a Poisson distribution with parameter 1, H∼ Poi(1). We then identify *H* segments with *H*–1 changepoints, $\tau =\{\tau_{1},\ldots ,\tau_{H-1}\}$, on the set S:={2,…,T−1}. Data point $D_{t}$ is assigned to the *h*th segment if and only if $\tau_{h-1}<t\leq \tau_{h}$, where $\tau_{0}:=1$ and $\tau_{H}:=T$. We follow Green (1995) and assume the changepoints to be distributed like the even-numbered order statistics of L:=2(H−1)+1 points, being uniformly distributed on S:

(13) $$p(\tau |H)=\frac{1}{\left(\begin{array}{c} T-2\\ 2(H-1)+1 \end{array}\right)}\cdot \prod_{h=0}^{H-1}\left(\tau_{h+1}-\tau_{h}-1\right)$$

Given π and τ, the model from Section 2.1 can be applied. The changepoint set τ yields a segmentation of the data into *H* segments with the response vector set $y_{\tau }:=\{y_{1},\ldots ,y_{H}\}$. The corresponding design matrices $X_{1},\ldots ,X_{H}$ are built using the values of the covariates in π. The parameters $\sigma^{2},\;\beta_{1},\ldots ,\beta_{H}$, *λ_u_*, *λ_c_* and the elements of δ can then be re-sampled from their FCDs; see Section 2.2. Given instantiations of *λ_u_*, *λ_c_* and δ, Metropolis-Hastings moves on π and τ can be designed. For each combination of covariate set π and changepoint set τ we can employ Equation (11) to compute the marginal likelihood. We get:

$$\begin{array}{c} p(\pi ,\tau ,\lambda_{u},\lambda_{c},\delta |D)\propto \\ p(y_{\tau }|\lambda_{u},\lambda_{c},\delta ,\pi ,\tau )\cdot p(\pi )\cdot p(\tau |H)\cdot p(H)\cdot p(\delta )\cdot p(\lambda_{u})\cdot p(\lambda_{c}) \end{array}$$

For inference we implement a Reversible Jump Markov Chain Monte Carlo (RJMCMC) sampling scheme (Green, 1995). We use changepoint birth, death and re-allocation moves for sampling the changepoint set, τ, and we use covariate addition, deletion and exchange moves for sampling the covariate set, π. We refer to Supplementary Material Part B for the mathematical details and pseudo-code of the RJMCMC algorithm. We then use RJMCMC simulations to generate a sample from the posterior distribution $p(\pi ,\tau ,\lambda_{u},\lambda_{c},\delta |D)$. In each iteration we first re-sample the parameters $\sigma^{2},\;\beta_{1},\ldots ,\beta_{H}$, *λ_u_*, *λ_c_* and δ from their full conditional distributions (Gibbs sampling), before we perform Metropolis-Hastings moves on the covariate set π and on the changepoint set τ. This way, a sample ${\left(\pi^{\left(w\right)},\tau^{\left(w\right)},\lambda_{u}^{\left(w\right)},\lambda_{c}^{\left(w\right)},\delta^{\left(w\right)}\right)}_{w=1,\ldots ,W}$ from the posterior distribution can be generated.

### 2.4 Learning dynamic networks via regression models

Consider a *N*-by-(T+1) data matrix D whose rows correspond to *N* network variables $Z_{1},\ldots ,Z_{N}$ and whose columns correspond to equidistant time points t=1,…,T+1. Let $D_{i,t}$ denote the value of *Z_i_* at *t*. The variables can then be identified with the nodes of a network, and we can learn how the variables interact with each other. Temporal data are conventionally modelled with dynamic Bayesian networks (DBNs), where all dependencies are subject to a time lag. An edge $Z_{i}\to Z_{j}$ indicates that $D_{j,t+1}$ (*Z_j_* at *t *+* *1) depends on $D_{i,t}$ (*Z_i_* at *t*).

Because of this time lag, there is no acyclicity constraint in DBNs, so that (piece-wise) linear regression can be applied *N* times independently. In the *j*th linear regression model $Y:=Z_{j}$ is the response, and there are n:=N−1 potential covariates: $\left\{X_{1},\ldots ,X_{n}\}:=\{Z_{1},\ldots ,Z_{j-1},Z_{j+1},\ldots ,Z_{N}\right\}$. Each data point $D_{t}$ (t=1,…,T) contains a response value $D_{j,t+1}$ and the shifted values $D_{1,t},\ldots ,D_{j-1,t},D_{j+1,t},\ldots ,D_{N,t}$ of the covariates.

The *N* individual covariate sets $\pi_{1},\ldots ,\pi_{N}$ for the responses $Z_{1},\ldots ,Z_{N}$ then describe a network: $G:=\{\pi_{1},\ldots ,\pi_{N}\}$. There is an edge from *Z_i_* to *Z_j_* if and only if $Z_{i}\in \pi_{j}$.

### 2.5 Network reconstruction

For each network variable *Z_j_* (j=1,…,N) we generate a posterior sample ${\left(\pi_{j}^{\left(w\right)},\tau_{j}^{\left(w\right)},\lambda_{u,j}^{\left(w\right)},\lambda_{c,j}^{\left(w\right)},\delta_{j}^{\left(w\right)}\right)}_{w=1,\ldots ,W}$; see Section 2.3. We then merge the sampled covariate sets to form a sample of graphs ${\left\{G^{\left(w\right)}\right\}}_{w=1,\ldots ,W}$, where $G^{\left(w\right)}:=(\pi_{1}^{\left(w\right)},\ldots ,\pi_{N}^{\left(w\right)})$. The *w*th graph $G^{\left(w\right)}$ has the edge $Z_{i}\to Z_{j}$ if $Z_{i}\in \pi_{j}^{\left(w\right)}$. For each edge $Z_{i}\to Z_{j}$ we compute the marginal edge posterior probability (score):

(14) $${\hat{e}}_{i,j}=\frac{1}{W}\sum_{w=1}^{W}I_{i\to j}(G^{\left(w\right)})$$

where $I_{i\to j}(G^{\left(w\right)})=1$ if $Z_{i}\in \pi_{j}^{\left(w\right)}$, and $I_{i\to j}(G^{\left(w\right)})=0$, otherwise.

If the true network is known, we evaluate the network reconstruction accuracy with precision-recall curves. For each ψ∈[0,1] we extract the n(ψ) edges whose scores ${\hat{e}}_{i,j}$ exceed *ψ*, and we count the number of true positives T(ψ) among them. Plotting the *precisions* P(ψ):=T(ψ)/n(ψ) against the *recalls* R(ψ):=T(ψ)/M, where *M* is the number of edges in the true network, gives the precision-recall curve. We refer to the area under the curve as AUC value.

The RJMCMC convergence can be monitored in terms of potential scale reduction factors (PSRFs); see, e.g. Brooks and Gelman (1998). On each dataset we perform *H *=* *10 independent RJMCMC simulations we monitor the fraction of edges that fulfilled *PSRF* < 1.01. For some convergence diagnostics we refer to Supplementary Material Part C.

### 2.6 Related sequentially coupled NH-DBN models

**Fig. 2.**
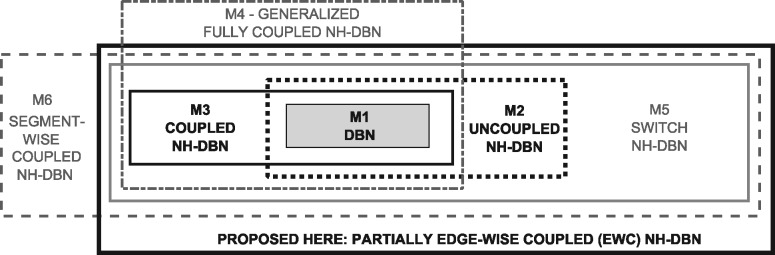
Overview of the NH-DBNs from Section 2.6. For each model there is a plate that covers the plates of the models that are nested (as special cases) within it

We outline six alternative regression models, with which NH-DBNs can be built. Like the EWC model, the models can be applied to each variable separately to infer a network. The last two models M5-M6 have not been proposed in the literature yet. We propose them here as competitors. For a graphical overview, on how the models are related (see Fig. 2).

- **M1: (HOMOGENEOUS) DBN** The conventional homogenous DBN, as discussed in many textbooks, has no changepoints, *H* = 1. The regression coefficient vector $\beta_{1}$ applies to all data points.
- **M2: (FULLY) UNCOUPLED NH-DBN** This model is akin to the model of Lèbre *et al.* (2010), but we here do not allow the network structure to be segment-specific. The EWC NH-DBN reduces to an uncoupled model for δ=0. The priors are: $\beta_{h}|(\sigma^{2},\lambda_{u})∼N(0,\sigma^{2}\lambda_{u}I)$ for all *h*.
- **M3: (FULLY) COUPLED NH-DBN** The M3 model from Grzegorczyk and Husmeier (2012) couples **all** neighbouring regression coefficients with the same strength. The EWC NH-DBN reduces to the coupled model when setting δ=1. The priors of the regression coefficients are: $\beta_{1}|(\sigma^{2},\lambda_{u})∼N(0,\sigma^{2}\lambda_{u}I)$ and $\beta_{h}|(\sigma^{2},\lambda_{c},{\tilde{\beta }}_{h-1})∼N({\tilde{\beta }}_{h-1},\sigma^{2}\lambda_{c}I)$ for h≥2.
- **M4: GENERALIZED (FULLY) COUPLED NH-DBN** The M4 model from Shafiee Kamalabad and Grzegorczyk (2018) generalizes the coupled NH-DBN (M3). It introduces segment-specific coupling strength parameters $\lambda_{c}^{h}$ (h=2,…,H):$\beta_{h}|(\sigma^{2},\lambda_{c}^{h},{\tilde{\beta }}_{h-1})∼\{\begin{array}{l} N(0,\;\;\;\;\;\;\;\lambda_{u}\sigma^{2}I)\;\text{if}\;h=1\\ N({\tilde{\beta }}_{h-1},\;\lambda_{c}^{h}\sigma^{2}I)\;\text{if}\;h>1 \end{array}$where $\lambda_{c}^{1}:=\lambda_{u}$, and $\lambda_{c}^{h}∼\mathrm{GAM}(a_{c},b_{c})$ for h=2,…,H. The coupling applies to *all* regression coefficients, but the coupling strengths $\lambda_{c}^{2},\ldots ,\lambda_{c}^{H}$ are segment-specific.
- **M5: SWITCH NH-DBN** The M5 model switches between an uncoupled and a coupled NH-DBN; i.e. it switches between the models M2 and M3.$\beta_{h}|(\sigma^{2},\ldots ,\delta^{*})∼\{\begin{array}{l} N(0,\;\;\;\;\;\;\;\lambda_{u}\sigma^{2}I)\;\text{if}\;\delta^{*}=0\;\;\;\text{or}\;\;\;h=1\\ N({\tilde{\beta }}_{h-1},\;\lambda_{c}\sigma^{2}I)\;\text{if}\;\delta^{*}=1\;\;\text{and}\;\;h>1 \end{array}$where $\delta^{*}∼\mathrm{BER}(0.5)$. It switches between coupled ($\delta^{*}=1$) and uncoupled ($\delta^{*}=0$). But coupling/uncoupling is *not* edge-wise. It applies to *all* regression coefficients, as if the EWC NH-DBN could switch only between the limiting states δ=0 and δ=1
- **M6: PARTIALLY SEGMENT-WISE COUPLED NH-DBN** The M6 model replaces the edge-wise by a segment-wise coupling concept. The model infers for each segment *h* > 1 if it is uncoupled from or coupled to the preceding segment. Coupling (uncoupling) then applies to *all* covariates simultaneously. The priors are:$\beta_{h}|(\sigma^{2},\ldots ,\delta_{h}^{*})∼\{\begin{array}{l} N(0,\;\;\;\;\;\;\;\;\;\lambda_{u}\sigma^{2}I)\;\text{if}\;\delta_{h}^{*}=0\;\;\;\text{or}\;\;\;h=1\\ N({\tilde{\beta }}_{h-1},\;\lambda_{c}\sigma^{2}I)\;\text{if}\;\delta_{h}^{*}=1\;\;\text{and}\;\;h>1 \end{array}$where $\delta_{1}^{*}:=0$, and $\delta_{h}^{*}∼\mathrm{BER}(0.5)$ for *h *>* *1. $\delta_{h}^{*}=1$ indicates that segment *h* is coupled to segment *h* – 1, while $\delta_{h}^{*}=0$ indicates that it is uncoupled. At each changepoint *all* regression coefficients stay either similar ($\delta_{h}^{*}=1$) or not ($\delta_{h}^{*}=0$). The underlying information-coupling scheme is thus not edge-wise but segment-wise.

### 2.7 Alternative network reconstruction models

We also include some alternative network reconstruction methods. Like the NH-DBN models M1-M6, the models A1-A7 can also be applied to each variable separately to infer a network.

- **A1: DBN + TRAFO** Like the DBN (M1), but we include covariate transformations. Given the covariates $\pi =\{X_{1},\ldots ,X_{k}\}$, we add all quadratic $X_{i}^{2}$ and all interaction $X_{i}X_{j}$ (i≠j) terms to the design matrix. We note that the idea is adopted from Oates and Mukherjee (2012).
- **A2: NH-DBN + TRAFO** Like the uncoupled NH-DBN (M2), but we add quadratic and interaction terms to the segment-specific design matrices; see A1.
- **A3: TESLA** TESLA is based on segment-specific L1-regularized linear regression and uses a second L1-regularization term to penalize dissimilarities between the regression coefficients of neighbouring segments. It can be seen as the frequentist counterpart of the coupled NH-DBN (M3). Inference is based on a penalized maximum likelihood approach, and the two regularization parameters have to be optimized. We apply 10-fold cross-validation (CV) with fine grids for the penalty parameters (0,0.01,…,1). TESLA is the only method in our comparison that allows the network structure to change over time. For our simulations we use the Matlab software from Kolar *et al.* (2010).
- **A4: HMM NH-DBN** This model from Grzegorczyk (2016) uses the priors of the uncoupled NH-DBN (M2), $\beta_{h}∼N(0,\sigma^{2}\lambda_{u}I)$, but unlike the M2 model it employs a more flexible hidden Markov model (HMM) to allocate the individual data points to the *H* components. For our simulations we use the Matlab software from Grzegorczyk (2016). The following methods A5-A7 use the concept of gradient matching. For each gene, the gradients (temporal derivatives) are estimated (e.g. via finite differences) and then used as response values within non-linear models.
- **A5: CHEMA** The CHEMA model from Oates *et al.* (2014) is a Bayesian model that employs differential equations, representing Michaelis-Menten kinetics, to explain the estimated gradients. For each response, the marginal likelihoods of all possible covariate sets are approximated, and the edge scores are obtained by marginalization over all covariate sets. We apply CHEMA in its improved variant (Aderhold *et al.*, 2017) and use thermodynamic integration with 25 inverse temperatures for approximating the marginal likelihoods. For our simulations we use the Matlab software from Aderhold *et al.* (2017).
- **A6: GP4GRN** The GP4GRN method from Äijö and Lähdesmäki (2009) is a Bayesian model that uses Gaussian Process (GP) regression with a Matérn class kernel to explain the gradients. For each response, the marginal likelihoods of all possible covariate sets are computed and the edge scores are obtained by marginalization over all covariate sets. For each covariate set the model hyperparameters (kernel parameters) are optimized with the Polack-Ribiere conjugate gradient method to maximize the marginal likelihood. For our simulations we use the Matlab software from Äijö and Lähdesmäki (2009).
- **A7: NeRDS** This method from Henderson and Michailidis (2014) is a frequentist model that uses smoothing-splines (rather than finite differences) to estimate the gradients. It then explains the gradients by a non-parametric additive model. Inference is based on sparse back-fitting, where univariate smoothing-splines are successively fitted to the estimated gradients. For our simulations we use the R software from Henderson and Michailidis (2014).

## 3 Implementation

For the inverse Gamma distributed parameters $\sigma^{2}$, *λ_u_* and *λ_c_* we select the shape and rate parameters: $a_{\sigma }=b_{\sigma }=0.005,\;a_{c}=a_{u}=2$ and $b_{c}=b_{u}=0.2$, as in earlier works (Grzegorczyk and Husmeier, 2012; Lèbre *et al.*, 2010). Pre-simulations with different settings showed robustness w.r.t. those hyperparameters. To ensure a fair comparison we use the same hyperparameters for the competing NH-DBNs.

For the NH-DBNs we ruled out (autoregressive) self-loops, such as $Z_{i}\to Z_{i}$, so as to be consistent with earlier studies (Grzegorczyk and Husmeier, 2012; Grzegorczyk, 2016; Lèbre *et al.*, 2010). Another reason is that self-loops can have negative effects on the network reconstruction accuracy, as empirically shown in Supplementary Material Part D.

For generating posterior samples, we run the RJMCMC algorithm for 100 000 (100k) iterations. We set the burn-in phase to 50k and we sampled every 100th graph during the sampling phase. We used potential scale reduction factors (PSRFs) to monitor convergence. For all datasets all PSRF’s were below 1.01 after 100k iterations; see Supplementary Figure S1 in Supplementary Material Part C for two examples. The computational costs for 100*k* RJMCMC iterations are relatively low when a modern computer cluster can be used. The task to infer a network with *N* nodes can be subdivided into *N* independent regression tasks (cf. Section 2.4), so that *N* simulations can run in parallel. With our Matlab implementation for each regression model 100k iterations took a few minutes.

A detailed analysis of the computational costs is provided in Supplementary Material Part E. The main finding is that also networks with *N *=* *100 nodes can be inferred with satisfactory convergence rate when 6–12 h of computational time are invested. On a computer cluster the network inference task can be separated into *N *=* *100 regression tasks, each taking 3.6–7.2 min of computational time. It is impossible to give a concrete upper bound on the maximal network size that can be inferred with the EWC NH-DBN. Proper Bayesian model inference requires that the RJMCMC sampling algorithm converges, and the convergence rate strongly depends on the posterior landscape. For landscapes with many local optimal regions, convergence can be slowed down, so that even small network inference might become challenging. On the other hand, even for large networks the RJMCMC algorithm might converge rather quickly (e.g. when the posterior landscape is unimodal).

## 4 Data

### 4.1 Synthetic network data

The RAF pathway, as reported in Sachs *et al.* (2005), has *N *=* *11 nodes and *M *=* *20 edges. We generate data consisting of *H *=* *4 segments with *m* data points each. For every node *Z_i_* (i=1,…,11) the parent nodes build the covariate set $\pi_{i}$ of the piece-wise linear regression model:

$$z_{i,t+1}=\beta_{i,F(t),0}+\sum_{j:Z_{j}\in \pi_{i}}\beta_{i,F(t),j}\cdot z_{j,t}+e_{i,t}\;\;\;\;(t=1,\ldots ,4m)$$

where $z_{k,t}$ denotes the value of node *Z_k_* at time point *t*. We sample the noise values $e_{i,t}$ and the initial values $z_{i,1}$ from independent $N(0,0.05^{2})$ distributions. The regression coefficients are subject to temporal changes, and change after *m* data points, so that F(t)=1+⌊(t−1)/m⌋. For each node *Z_i_* there are $|\pi_{i}|+1$ regression coefficients with *H *=* *4 segment-specific values. For each segment *h* we summarize the $|\pi_{i}|+1$ coefficients for response *Z_i_* in a vector $\beta_{i,h}={\left(\beta_{i,h,0},\ldots ,\beta_{i,h,|\pi_{i}|}\right)}^{T}$.

We sample the elements of $\beta_{i,h}$ (h=1,…,4) from standard *N*(0, 1) Gaussian distributions and then normalize the vectors to Euclidean norm one, i.e. for h=1,…,4: $\beta_{i,h}\leftarrow \beta_{i,h}/|\beta_{i,h}|$. We distinguish four regression coefficient types. The four regression coefficients $\beta_{i,1,j},\ldots ,\beta_{i,4,j}$ for the edge $Z_{j}\to Z_{i}$ can be:

1. **‘coupled’:** We keep the regression coefficient fixed among segments. To this end, we replace: $\beta_{i,h,j}\leftarrow \beta_{i,1,j}$ (*h* = 2, 3, 4).
2. **‘similar’:** We enforce the four segment-specific regression coefficients to have the same sign, i.e. we replace $\beta_{i,h,j}\leftarrow \text{sign}(\beta_{i,1,j})\cdot |\beta_{i,h,j}|$.
3. **‘independent’:** We leave the four independent segment-specific regression coefficients $\beta_{i,1,j},\ldots ,\beta_{i,4,j}$ unchanged.
4. **‘dissimilar’:** We enforce the segment-specific regression coefficients to change the sign, i.e. we set $\beta_{i,h,j}\leftarrow \text{sign}(-\beta_{i,h-1,j})\cdot |\beta_{i,h,j}|$.

The RAF network has $\sum_{i=1}^{11}\left(|\pi_{i}|+1\right)=31$ regression coefficients. We assume that *K* randomly selected regression coefficients are ‘coupled’, while all the remaining ones are either ‘similar’, or ‘independent’ or ‘dissimilar’. This yields three different scenarios (mixtures of T1&T2, T1&T3 and T1&T4). For K∈{0,3,…,27,31} we obtain different percentages of coupled edges. For each scenario and every *K* we generate 100 independent datasets with different regression coefficients and *m *=* *5 data points per segment (3300 datasets in total). To each dataset we add observational noise using a signal-to-noise ratio of 3. For each node *Z_i_* we compute the standard deviation *s_i_* of its values $z_{i,1},\ldots ,z_{i,4m+1}$, and we then add to each $z_{i,j}$ the realization of a $N(0,{\left(s_{i}/3\right)}^{2})$ distribution.

### 4.2 Yeast gene expression data

By means of synthetic biology, Cantone *et al.* (2009) designed a network with in *S.cerevisiae* (yeast). With Real-Time Polymerase Chain Reaction (RT-PCR), Cantone et al. then measured in vivo gene expression data: first under galactose- and then under glucose-metabolism. For both carbon sources the network structure is identical (Cantone *et al.*, 2009), but the strengths of the regulatory processes change with the carbon source (Cantone *et al.*, 2009); 16 (19) measurements were taken in galactose (glucose). We provide more details in Supplementary Material Part C.

### 4.3 Arabidopsis gene expression data

The circadian clock in *A.thaliana* synchronizes the plant metabolism with the 24-h photo period. The circadian clock can anticipate the photo period and optimize the regulatory processes. The network structure does not change, but the gene interaction strengths depend on the external (or entrained) photo periods. See, e.g. Figure 12 in Aderhold *et al.* (2014) for an overview of time-invariant network structure hypotheses from different authors. In each network structure hypothesis the effect of light is indicated by a ‘sun’ symbol. In four experiments Arabidopsis plants were entrained in different dark: light cycles, before data were collected every 2 or 4 h under constant light. We focus on the core clock genes, and we merge the data into one time series; for details see Supplementary Material Part C.

## 5 Empirical results

### 5.1 Results on synthetic RAF-pathway data

We use the synthetic RAF pathway data to compare the performance of the EWC NH-DBN with: M1 (DBN), M2 (UNCOUPLED NH-DBN), M3 (COUPLED NH-DBN), A1 (DBN+TRAFO) and A3 (TESLA).

The gradient-based models (A6–A8) are not suitable here, as the data generation (Section 4.2) does not yield meaningful functional relationships in the individual temporal profiles. To avoid that the NH-DBNs reduce to a DBN (without changepoints) when the percentages of coupled edges approach 100%, we assume the changepoints to be known, so that the changepoints do not have to be inferred from the data.

**Fig. 3.**
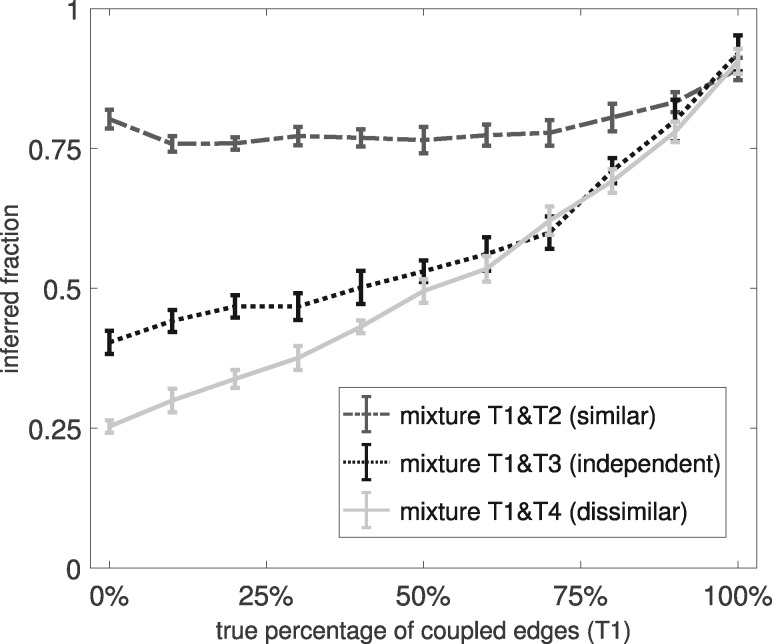
Diagnostics for the EWC NH-DBN. For three mixture scenarios and 11 different percentages of coupled edges we computed the inferred average fraction of coupled edges. We then plotted the average fractions against the true percentages

Figure 3 shows the fractions of coupled edges that the EWC NH-DBN inferred. The trends are in agreement with the true data generation processes. The fractions increase with the true percentages, and the fractions are scenario-dependent. When the non-coupled edges are ‘similar’ (T2) or ‘dissimilar’ (T4), the inferred fractions are higher or lower, respectively. Overall, the inferred fractions of coupled edges tend to be higher than the true fractions. That is, there is a certain bias towards coupling too many edges. In particular, this applies to scenario T1&T2, where even the non-coupled edges have similar regression coefficients. Here the inferred fractions are consistently too high. For the other two scenarios the bias gets weaker as the true percentage increases.

**Fig. 4.**
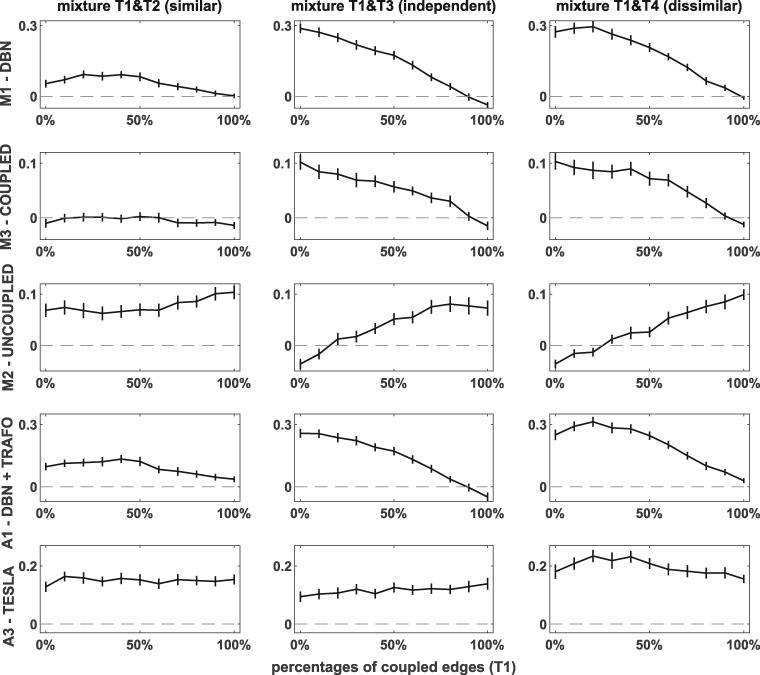
Relative AUC differences in favour of the EWC NH-DBN (RAF pathway data). We consider three scenarios (mixtures of T1&T2, T1&T3 and T1&T4) with varying percentages of coupled edges (T1). The columns refer to the three scenarios and each rows refers to a competing model. In the panels the AUC differences (averaged across 100 datasets) have been plotted against the percentage of coupled edges. The error bars on the curve correspond to 0.95 confidence intervals of paired two-sample t-tests

Figure 4 shows the relative AUC differences in favour of the EWC NH-DBN. For the average AUC values we refer to Supplementary Material Part F. From Figure 4 the following trends (i-iv) can be observed:

- **EWC NH-DBN v** **ersu** **s** **DBN (M1) and versus** **DBN+TRAFO (A1)** The 1st and 4th row show that the quadratic/interaction terms do not lead to improvements. This is consistent with the results in Oates and Mukherjee (2012). The superiority of the EWC NH-DBN over the homogeneous DBNs diminishes as the percentage of coupled edges increases. Although the superiority is significant, the AUCs are only slightly increased for large percentages (>50%) of coupled edges. Except for scenario ‘T1&T2’, where even the non-coupled regression coefficients stay similar, the AUC improvement is substantial (> 0.18 and >0.20) when the percentage of coupled edges is low (≤50%).
- **EWC NH-DBN v** **ersu** **s** **coupled NH-DBN (M3)** The trend in the 2nd row is similar to case (i). For scenario ‘T1&T2’ both models perform approximately equally well, but for high percentages of coupled edges (≥70%) the EWC NH-DBN is slightly inferior. Like for the DBNs, for the other two scenarios the superiority of the EWC NH-DBN diminishes as the percentage of coupled edges increases. The AUC improvements for small percentages (≤50%) are moderate (0.08−0.10).
- **EWC NH-DBN v** **ersu** **s** **uncoupled NH-DBN (M2)** The 2nd row shows an opposite trend: The uncoupled NH-DBN is consistently outperformed for scenario ‘T1&T2’, though the AUC improvement is only moderate (≈0.08). For the other two scenarios the superiority of the EWC NH-DBN model rises as the percentage of coupled edges increases. The AUC improvements for large percentages (≥50%) are again moderate (0.04−0.09). Here the EWC NH-DBN is slightly inferior when the percentage of coupled edges is very low (≤10%)
- **EWC NH-DBN v** **ersu** **s** **TESLA (A3)** The 5th row shows that TESLA is consistently inferior to the EWC NH-DBN. This result is consistent with the result of the cross-method comparison of Aderhold *et al.* (2014), where TESLA was also found to perform below average. Diagnostics (not shown) revealed that TESLA sometimes inferred different network structure for the segments. We note that TESLA is the only method in the comparison that allows for time-varying network structures; a feature that is not required here.

### 5.2 Results on yeast gene expression data

**Fig. 5.**
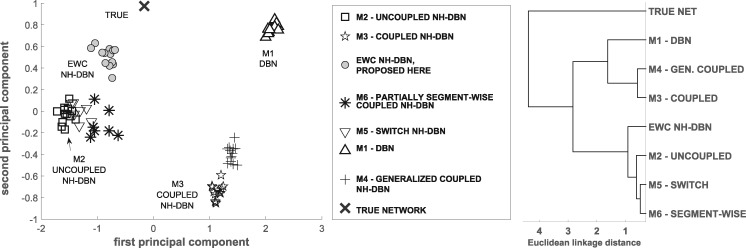
Yeast data: PCA and dendrogram plot of the edge scores of the sequentially coupled NH-DBNs from Section 2.6. Every RJMCMC simulation outputs edge scores ${\hat{e}}_{i,j}$ for all edges. We arrange the scores of each individual simulation vector-wise and standardize all vectors (to mean 0 and variance 1). **Left:** Standard PCA plot to project the set of vectors onto the first two principal components, explaining 78%+10% of the variance. **Right:** For each model we then averaged the score vectors across the simulations and clustered the model-specific average vectors based on their Euclidean distances. The dendrogram shows the results

As the yeast network is known, we can cross-compare the network reconstruction accuracies on real in vivo gene expression data. For each of the NH-DBNs from Section 2.6 we run *H *=* *10 independent RJMCMC simulations. Each simulation yields an edge score ${\hat{e}}_{i,j}$ for each potential edge. We arrange the simulation-specific scores in vectors $v_{m,h}$, where *m* indicates the NH-DBN model and *h* the simulation. In addition we build the true vector $v^{*}$ whose entries are 1 if the corresponding edge is present, or 0 otherwise. We propose to use a principal component analysis (PCA) and a cluster analysis to visualize (dis-)similarities between the NH-DBNs from Section 2.6. To this end, we zscore-standardize all vectors, and project them onto the first two principal components (PCs). Figure 5 shows the resulting PCA plot and a dendrogram of the model-specific average score vectors. For the dendrogram we clustered the model-specific average score vectors based on their Euclidean distances. The first two PCs explain 78 and 10% (together ≈90%) of the variance, so that the 2-dimensional PCA plot conserves most of the information. Taking into account that the 1st PC ($\lambda_{1}=1.94$) has more weight than the 2nd PC ($\lambda_{2}=0.24$), the following trends can be seen: (i) The model-specific simulations are always closely grouped together, i.e. independent simulations yield similar edge scores, what is a good indicator for convergence. (ii) Nearest to the true network is the new EWC NH-DBN, while the DBN (M1) has the furthest distance. The partially segment-wise coupled model (M6) is 2nd nearest to the true network. (iii) The coupled model (M3) and its generalization with segment-specific coupling strengths (M4) yield similar edge scores, so that this improvement has a minor effect here. (iv) The points of the switch (M5) and the partially coupled NH-DBN (M6) are near to the uncoupled NH-DBN (M2). We conclude that M5 and M6 infer the majority of genes/segments to be uncoupled. The dendrogram shows that there are two model clusters. In the first cluster, the coupled NH-DBNs, which enforce coupling, group with the DBN. In the second cluster, the more flexible NH-DBNs, which have mechanisms to uncouple, group with the uncoupled NH-DBN.

**Fig. 6.**
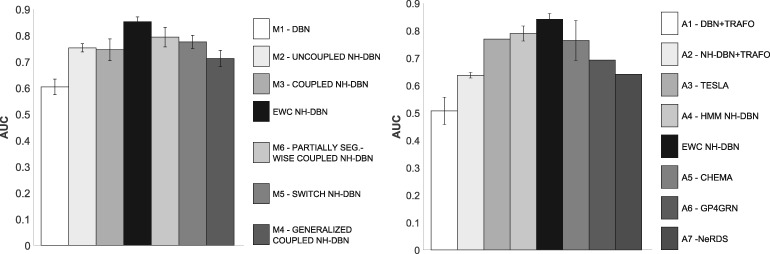
Network reconstruction accuracy for yeast. The two histograms compare the AUCs of the proposed EWC NH-DBN with the AUCs of the NH-DBN models from Section 2.6 (left histogram) and the AUCs of the competing methods from Section 2.7 (right histogram). For models that are inferred by MCMC techniques, the error bars indicate standard deviations

Figure 6 shows the network reconstruction accuracies of the EWC NH-DBN and the related NH-DBNs from Section 2.6 in terms of average AUC values. The EWC NH-DBN, which has the minimal distance to the true network in the PCA plot, yields the highest AUC value. More generally, the AUC values consistently decrease with the distance to the true network in the PCA plot, so that the AUC values and the PCA plot are in agreement. We performed two-sided unpaired t-tests and found that the average AUC of the EWC NH-DBN is significantly higher than the AUC of any other method (all six *P*-values: < 0.05). The right histogram in Figure 6 compares the AUC of the EWC NH-DBN with the AUCs of the models from Section 2.7. Again the EWC NH-DBN reaches the highest AUC score and two-sided unpaired t-tests indicate that the improvement is significant (all seven *P*-values: <0.05). In Supplementary Material Part G we provide more results, including the pairwise AUC differences.

### 5.3 Results on Arabidopsis gene expression data

**Fig. 7.**
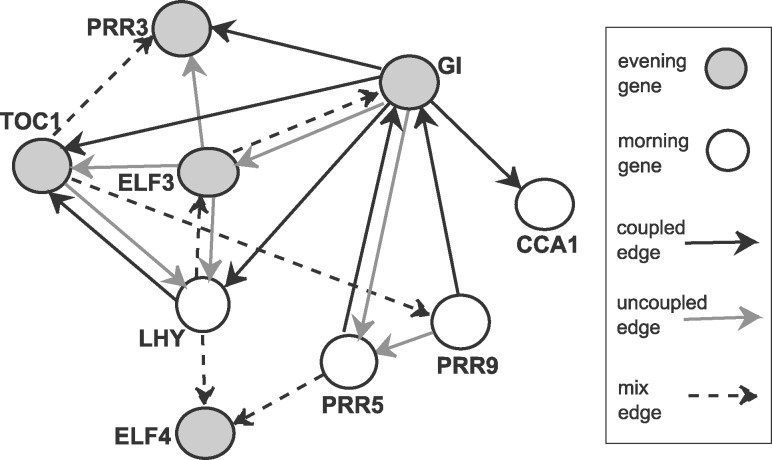
Predicted Arabidopsis network. Morning (evening) genes are represented as white (grey) nodes. We extracted the 20 edges with the highest scores. Different edge types indicate whether the parameters are coupled, uncoupled, or a mixture thereof. A coupled (uncoupled) edge indicates that the gene interaction is likely to be influenced (not affected) by light

The absence of a gold standard for the circadian clock network renders an objective evaluation of the network reconstruction accuracy impossible. We therefore focus on the EWC NH-DBN and illustrate that this model yields more insight into the robustness of the individual gene interactions. We run *H *=* *10 RJMCMC simulations and average the edge scores. For the posterior probabilities of the changepoint location we refer to Supplementary Material Part H. Onto the scores we impose a threshold *ψ* such that the 20 edges with the highest scores are extracted; the corresponding threshold is around ψ=2/3. Recalling that ${\hat{e}}_{i,j}$ refers to an edge from *X_i_* to *Y* = *Z_j_*, we consider the corresponding sampled *δ_i_* indicator variables and estimate the posterior probabilities that the edge was mainly ’coupled’ (or ’uncoupled’). If the posterior probability $\hat{p}(\delta_{i}=1|D)$ of the state ’coupled’ was double as likely as the probability $\hat{p}(\delta_{i}=0|D)$ of the state ’uncoupled’, we call the edge a ’coupled’ edge. Correspondingly, we call the edge ‘uncoupled’ if $p(\delta_{i}=0|D)>2\cdot p(\delta_{i}=1|D)$, and we call the edge ’mix edge’ if none of the conditions is satisfied. Figure 7 shows the predicted network with different symbols for the edge types. In this application to the circadian clock in Arabidopsis, the edge label (coupled versus uncoupled) can be interpreted as an indicator whether the corresponding gene interaction is likely to be light-dependent (=uncoupled) or not (=coupled).

In the biological literature, we could find evidence for some features of our network. The important key feature of the circadian clock network is the feedback loop between *LHY* and *TOC*1. This feedback is already known since Locke *et al.* (2006) and plays a central role in circadian regulation [see also more recent works, e.g. Pokhilko *et al.* (2013)]. The EWC NH-DBN does not only infer this feedback loop but also suggests that the effect of *LHY* on *TOC*1 is not light-dependent, while the regulatory effect of *TOC*1 on *LHY* appears to depend on light. Focusing on those two genes, we further found the following: The regulatory effect of *ELF*3 on *TOC*1, e.g. reported in Miwa *et al.* (2006), is also light-dependent, while the edge from *GI* to *TOC*1, also reported in Miwa *et al.* (2006), is not. The edges from *ELF*3 to *LHY* and from *LHY* to *ELF*4 have been reported in Kikis *et al.* (2005). The model finds both edges and provides evidence that the effect of *ELF*3 on *LHY* depends on the presence of light. For the effects of TOC1 on the *PRR*3 and *PRR*9 (Pokhilko *et al.*, 2013) the EWC NH-DBN switches between both labels (coupled and uncoupled), so that it stays unclear whether those two interactions are light-dependent.

## 6 Conclusions

We have proposed a non-homogeneous dynamic Bayesian network (NH-DBN) with an edge-wise coupling (EWC) scheme for the interaction parameters. Unlike earlier proposed NH-DBNs, the EWC NH-DBN infers for each individual edge whether its interaction parameter should be coupled (=stays similar over time) or better stay uncoupled (=changes in time). In some biological applications this insight into the robustness of the network interactions could be very useful.

Our results on a benchmark yeast gene expression data have shown that the EWC NH-DBN reaches a higher network reconstruction accuracy than thirteen state-of-the-art models. For the circadian clock in *A.thaliana* the EWC NH-DBN learned a plausible network structure and also indicated which of the gene interactions are likely to be light-dependent.

The proposed ‘edge-wise coupling’ (EWC) concept is generic, and could also be implemented for NH-DBNs with time-varying network structures. The coupled model (Grzegorczyk and Husmeier, 2012) cannot be applied, as the covariate sets vary from segment to segment. Under the condition that parameters associated with non-omnipresent edges have to stay ‘uncoupled’, the edge-wise coupling scheme could be directly adopted.

An idea for future research would be to generalize the coupled NH-DBN by introducing edge-specific coupling parameters, as suggested by one of the reviewers of this paper. Every edge-specific binary variable $\delta_{i}\in \{0,1\}$ would then be replaced by a continuous coupling strengths $\lambda_{c,i}\in R^{+}$. As edge additions/deletions change the number of $\lambda_{c,i}$ variables, the new model would have changing numbers of continuous variables. Hence, the main challenge would be to design efficient trans-dimensional RJMCMC moves in *continuous* parameter spaces (Green, 1995).

Another auspicious direction of future research would be to develop non-homogeneous versions of the Bayesian non-linear models, such as CHEMA and Gp4GRN, by combining them with a multiple changepoint process. This could, in principle, be done along the same lines that DBNs have been extended to non-homogeneous DBNs (NH-DBNs).

## Supplementary Material

btz690_Supplementary_Data
