# Supplementary material for: Non-homogeneous dynamic Bayesian networks with edge-wise sequentially coupled parameters
Source: Bioinformatics. 2019 Sep 5;36(4):1198–207. doi: 10.1093/bioinformatics/btz690 (PMC7703764; doi:10.1093/bioinformatics/btz690)
Supplement: btz690_Supplementary_Data [file btz690_supplementary_data.zip › SUPP_PAPER.pdf]

Supplementary material for the paper

‘Non-homogeneous dynamic Bayesian networks with edge-wise sequentially coupled parameters’

M. Shafiee Kamalabad and M. Grzegorczyk

Bioinformatics, 2019

Part A - Gibbs sampling (see Section 2.2 of the main paper)

The joint distribution of the new model can be factorized as follows:

$$\begin{aligned} p(\mathbf{y}_1, \ldots \mathbf{y}_H, \boldsymbol{\beta}_1, \ldots, \boldsymbol{\beta}_H, \sigma^2, \lambda_u, \lambda_c, \boldsymbol{\delta}) \quad &\propto \quad \left(\prod_{h=1}^{H} p(\mathbf{y}_h|\sigma^2, \boldsymbol{\beta}_h)\right) \cdot p(\lambda_u) \cdot p(\lambda_c) \cdot p(\sigma^2) \cdot p(\boldsymbol{\delta}) \\ &\cdot P(\boldsymbol{\beta}_1|\sigma^2, \lambda_u) \cdot \prod_{h=2}^{H} P(\boldsymbol{\beta}_h|\sigma^2, \lambda_u, \lambda_c, \boldsymbol{\delta}, \tilde{\boldsymbol{\beta}}_{h-1}) \end{aligned}$$

As bivariate function of λ_u and λ_c, the newly introduced prior:

$$\boldsymbol{\beta}_{h+1}|(\sigma^2, \lambda_u, \lambda_c, \tilde{\boldsymbol{\beta}}_h, \boldsymbol{\delta}) \sim \mathcal{N}(\boldsymbol{\delta} \odot \tilde{\boldsymbol{\beta}}_h, \sigma^2 \cdot diag\{\lambda_c \boldsymbol{\delta} + \lambda_u(\mathbf{1} - \boldsymbol{\delta})\})$$

has a modular form:

$$\begin{aligned} p(\boldsymbol{\beta}_{h+1}|\lambda_u, \lambda_c, \ldots) \quad &= \quad (2\pi)^{(-k+1)/2} \cdot \det\left(\sigma^2 \cdot diag\{\lambda_c \boldsymbol{\delta} + \lambda_u(\mathbf{1} - \boldsymbol{\delta})\}\right)^{-0.5} \\ &\cdot \exp\{-\frac{1}{2}(\boldsymbol{\beta}_{h+1} - \boldsymbol{\delta} \odot \tilde{\boldsymbol{\beta}}_h)^{\mathsf{T}}[\sigma^2 diag\{\lambda_c \boldsymbol{\delta} + \lambda_u(\mathbf{1} - \boldsymbol{\delta})\}]^{-1}(\boldsymbol{\beta}_{h+1} - \boldsymbol{\delta} \odot \tilde{\boldsymbol{\beta}}_h)\} \\ &= \quad (2\pi)^{-(k+1)/2} \cdot \sigma^{-(k+1)} \cdot \lambda_u^{-0.5\sum_{i=0}^{k}(1-\delta_i)} \cdot \lambda_c^{-0.5\sum_{i=0}^{k}\delta_i} \\ &\cdot \exp\{-\frac{1}{2}\sigma^{-2}\lambda_u^{-1} \sum_{i:\delta_i=0} (\beta_{h,i} - 0)^2\} \cdot \exp\{-\frac{1}{2}\sigma^{-2}\lambda_c^{-1} \sum_{i:\delta_i=1} (\beta_{h,i} - \tilde{\beta}_{h,i})^2\} \end{aligned}$$

As a function of λ_u^{-1} or λ_c^{-1}, respectively, the newly introduced prior, $p(\boldsymbol{\beta}_{h+1}|\lambda_u, \lambda_c, \ldots)$, is thus proportional to:

$$p(\boldsymbol{\beta}_{h+1}|\lambda_u, \ldots) \propto (\lambda_u^{-1})^{0.5\sum_{i=0}^{k}(1-\delta_i)} \cdot \exp\{-\lambda_u^{-1} \cdot (\frac{1}{2}\sigma^{-2} \cdot \sum_{i:\delta_i=0} \beta_{h,i}^2)\} \tag{1}$$

$$p(\boldsymbol{\beta}_{h+1}|\lambda_c, \ldots) \propto (\lambda_c^{-1})^{0.5\sum_{i=0}^{k}\delta_i} \cdot \exp\{-\lambda_c^{-1}(\frac{1}{2}\sigma^{-2} \cdot \sum_{i:\delta_i=1} (\beta_{h,i} - \tilde{\beta}_{h,i})^2)\} \tag{2}$$

The prior for $\boldsymbol{\beta}_1$, $\boldsymbol{\beta}_1 \sim \mathcal{N}(\mathbf{0}, \sigma^2\lambda_u\mathbf{I})$, is independent of λ_c. As a function of λ_u^{-1} we get:

$$p(\boldsymbol{\beta}_1|\sigma^2, \lambda_u, \boldsymbol{\delta}) \propto (\lambda_u^{-1})^{0.5(k+1)} \cdot \exp\{-\lambda_u^{-1} \cdot (\frac{1}{2}\sigma^{-2} \cdot \sum_{i=0}^{k} \beta_{1,i}^2)\} \tag{3}$$

When deriving the full conditional distributions of λ_u and λ_c, we make use of Equations (1-3).

Deriving the full conditional distribution

When deriving the full conditional distributions (FCDs), we make use of the fact that the full conditional densities are proportional to the factorized joint density.

Regression coefficient vectors:
For the regression coefficient vectors $\boldsymbol{\beta}_h$ we can apply standard rules (see, e.g., Chapters 2-3 of Bishop (2006)). For $h = 1$ we set $\boldsymbol{\mu}_1 := \mathbf{0}$ and $\boldsymbol{\Sigma}_1 := diag\{\lambda_u \mathbf{1}\}$. For $h > 1$ we set $\boldsymbol{\mu}_h := \boldsymbol{\delta} \odot \tilde{\boldsymbol{\beta}}_{h-1}$ and $\boldsymbol{\Sigma}_h := diag\{\lambda_c \boldsymbol{\delta} + \lambda_u(\mathbf{1} - \boldsymbol{\delta})\}$ so that all regression coefficient priors take the form:

$$\boldsymbol{\beta}_h | (\sigma^2, \boldsymbol{\mu}_h, \boldsymbol{\Sigma}_h) \sim \mathcal{N}(\boldsymbol{\mu}_h, \sigma^2 \cdot \boldsymbol{\Sigma}_h)$$

The rule from Section 3.3 of Bishop (2006) then implies:

$$FCD(\boldsymbol{\beta}_h) := \boldsymbol{\beta}_h | (\mathbf{y}_h, \sigma^2, \lambda_u, \lambda_c) \sim N(\tilde{\boldsymbol{\beta}}_h, \sigma^2 \mathbf{C}_h) \tag{4}$$

where $\mathbf{C}_h = (\boldsymbol{\Sigma}_h^{-1} + \mathbf{X}_h^\mathsf{T} \mathbf{X}_h)^{-1}$ and $\tilde{\boldsymbol{\beta}}_h = \mathbf{C}_h(\boldsymbol{\Sigma}_h^{-1} \boldsymbol{\mu}_h + \mathbf{X}_h^\mathsf{T} \mathbf{y}_h)$.

Noise variance parameter:
The noise variance parameter, σ^2, can be re-sampled via a collapsed Gibbs sampling step, where the regression coefficients, $\boldsymbol{\beta}_1, \ldots, \boldsymbol{\beta}_H$, have been integrated out. A standard rule for Gaussian integrals (see, e.g., Section 2.3.2 in Bishop (2006)) is that:

$$\mathbf{y} | \boldsymbol{\beta} \sim \mathcal{N}(\mathbf{X}\boldsymbol{\beta}, \boldsymbol{\Sigma}) \text{ with } \boldsymbol{\beta} \sim \mathcal{N}(\boldsymbol{\mu}, \mathbf{S})$$

implies $\mathbf{y} \sim \mathcal{N}(\mathbf{X}\boldsymbol{\mu}, \boldsymbol{\Sigma} + \mathbf{X}\mathbf{S}\mathbf{X}^\mathsf{T})$ for the marginal distribution with density $p(\mathbf{y}) = \int p(\mathbf{y}, \boldsymbol{\beta})\, d\boldsymbol{\beta}$.

We now use this rule for computing the marginal distribution of $\mathbf{y}_h$, marginalized over $\boldsymbol{\beta}_h$:

$$\begin{aligned} p(\mathbf{y}_h | \sigma^2, \lambda_u, \lambda_c, \boldsymbol{\delta}) &= \int p(\mathbf{y}_h, \boldsymbol{\beta}_h | \sigma^2, \lambda_u, \lambda_c, \boldsymbol{\delta})\, d\boldsymbol{\beta}_h \\ &= \int p(\mathbf{y}_h | \boldsymbol{\beta}_h, \sigma^2, \lambda_u, \lambda_c, \boldsymbol{\delta}) p(\boldsymbol{\beta}_h | \sigma^2, \lambda_u, \lambda_c, \boldsymbol{\delta})\, d\boldsymbol{\beta}_h \end{aligned}$$

With $\mathbf{y}_h | (\boldsymbol{\beta}_h, \sigma^2) \sim \mathcal{N}(\mathbf{X}_h \boldsymbol{\beta}_h, \sigma^2 \mathbf{I})$ and

$$\boldsymbol{\beta}_h | (\sigma^2, \lambda_u, \lambda_c, \tilde{\boldsymbol{\beta}}_h, \boldsymbol{\delta}) \sim \begin{cases} \mathcal{N}(\boldsymbol{\delta} \odot \tilde{\boldsymbol{\beta}}_0, & \sigma^2 diag\{\lambda_u \mathbf{1}\}) \quad \text{if } h = 1 \\ \mathcal{N}(\boldsymbol{\delta} \odot \tilde{\boldsymbol{\beta}}_{h-1}, & \sigma^2 diag\{\lambda_c \boldsymbol{\delta} + \lambda_u(\mathbf{1} - \boldsymbol{\delta})\}) \quad \text{if } h > 1 \end{cases}$$

where $\tilde{\boldsymbol{\beta}}_0 := \mathbf{0}$, so that $\boldsymbol{\delta} \odot \tilde{\boldsymbol{\beta}}_0 = \mathbf{0}$, the rule implies for the marginalisation over $\boldsymbol{\beta}_h$:

$$\mathbf{y}_h | (\sigma^{-2}, \lambda_u, \lambda_c, \boldsymbol{\delta}) \sim \begin{cases} \mathcal{N}(\mathbf{X}_1(\boldsymbol{\delta} \odot \tilde{\boldsymbol{\beta}}_0), & \sigma^2[\mathbf{I} + \mathbf{X}_1 diag\{\lambda_u \mathbf{1}\} \mathbf{X}_1^\mathsf{T}]) \quad \text{if } h = 1 \\ \mathcal{N}(\mathbf{X}_h(\boldsymbol{\delta} \odot \tilde{\boldsymbol{\beta}}_{h-1}), & \sigma^2[\mathbf{I} + \mathbf{X}_h diag\{\lambda_c \boldsymbol{\delta} + \lambda_u(\mathbf{1} - \boldsymbol{\delta})\} \mathbf{X}_h^\mathsf{T}]) \quad \text{if } h > 1 \end{cases}$$

As a function of σ^{-2} we have:

$$p(\mathbf{y}_h | \sigma^{-2}, \ldots) \propto (\sigma^{-2})^{T_h/2} \exp\{-\frac{1}{2} \sigma^{-2} (\mathbf{y}_h - \mathbf{X}_h(\boldsymbol{\delta} \odot \tilde{\boldsymbol{\beta}}_{h-1}))^\mathsf{T} (\mathbf{I} + \mathbf{X}_h \boldsymbol{\Sigma}_h \mathbf{X}_h^\mathsf{T})^{-1} (\mathbf{y}_h - \mathbf{X}_h(\boldsymbol{\delta} \odot \tilde{\boldsymbol{\beta}}_{h-1}))\}$$

where T_h is the length of the vector $\mathbf{y}_h$ and

$$\mathbf{\Sigma}_h := \begin{cases} diag\{\lambda_u \mathbf{1}\} & \text{if } h = 1 \\ diag\{\lambda_c \boldsymbol{\delta} + \lambda_u(\mathbf{1} - \boldsymbol{\delta})\} & \text{if } h > 1 \end{cases}$$

For the product of the segment-specific marginal likelihoods, $\prod p(\mathbf{y}_h|\sigma^{-2}, \lambda_u, \lambda_c, \boldsymbol{\delta})$, we get:
As a function of σ^{-2}:

$$\prod_{h=1}^{H} p(\mathbf{y}_h|\sigma^{-2}, \lambda_u, \lambda_c, \boldsymbol{\delta}) \propto (\sigma^{-2})^{0.5 \cdot T} \exp\{-0.5 \cdot \sigma^{-2} \cdot \Delta^2\}$$

where $T = \sum_{h=1}^{H} T_h$ is the number of data points, and

$$\Delta^2 := \sum_{h=1}^{H} (\mathbf{y}_h - \mathbf{X}_h(\boldsymbol{\delta} \odot \tilde{\boldsymbol{\beta}}_{h-1}))^{\mathsf{T}} (\mathbf{I} + \mathbf{X}_h \mathbf{\Sigma}_h \mathbf{X}_h^{\mathsf{T}})^{-1} (\mathbf{y}_h - \mathbf{X}_h(\boldsymbol{\delta} \odot \tilde{\boldsymbol{\beta}}_{h-1}))$$

with $\tilde{\boldsymbol{\beta}}_0 := \mathbf{0}$, and $\tilde{\boldsymbol{\beta}}_h = (\mathbf{\Sigma}_h^{-1} + \mathbf{X}_h^{\mathsf{T}} \mathbf{X}_h)^{-1} (\mathbf{\Sigma}_h^{-1} \boldsymbol{\mu}_h + \mathbf{X}_h^{\mathsf{T}} \mathbf{y}_h)$ being the posterior expectation of $\boldsymbol{\beta}_h$ ($h \geq 1$), given the prior expectations $\boldsymbol{\mu}_h$ and the prior covariance matrices $\mathbf{\Sigma}_h$:

$$\boldsymbol{\mu}_h = \begin{cases} \mathbf{0} & \text{if } h = 1 \\ \boldsymbol{\delta} \odot \tilde{\boldsymbol{\beta}}_{h-1} & \text{if } h > 1 \end{cases}, \qquad \mathbf{\Sigma}_h = \begin{cases} diag\{\lambda_u \mathbf{1}\} & \text{if } h = 1 \\ diag\{\lambda_c \boldsymbol{\delta} + \lambda_u(\mathbf{1} - \boldsymbol{\delta})\} & \text{if } h > 1 \end{cases}$$

Using this result, we obtain:

$$\begin{aligned} p(\sigma^{-2}|\mathbf{y}_1, \ldots, \mathbf{y}_H, \lambda_u, \lambda_c, \boldsymbol{\delta}) &\propto \left(\prod_{h=1}^{H} p(\mathbf{y}_h|\lambda_u, \lambda_c, \boldsymbol{\delta}, \boldsymbol{\pi}, \boldsymbol{\tau}) \right) \cdot p(\sigma^{-2}) \cdot p(\lambda_u) \cdot p(\lambda_c) \cdot p(\boldsymbol{\delta}) \\ &\propto (\sigma^{-2})^{0.5 \cdot T} \exp\{-0.5 \cdot \sigma^{-2} \cdot \Delta^2\} \cdot (\sigma^{-2})^{a_\sigma - 1} \exp\{-b_\sigma \sigma^{-2}\} \\ &\propto (\sigma^{-2})^{a_\sigma + 0.5 \cdot T - 1} \exp\{-\sigma^{-2}(b_\sigma + 0.5 \cdot \Delta^2)\} \end{aligned}$$

From the shape of the latter distribution it follows

$$FCD_C(\sigma^{-2}) := \sigma^{-2}|(\mathbf{y}_1, \ldots, \mathbf{y}_H, \lambda_u, \lambda_c, \boldsymbol{\delta}, \boldsymbol{\pi}, \boldsymbol{\tau}) \sim GAM\left(a_\sigma + 0.5 \cdot T, b_\sigma + 0.5 \cdot \Delta^2\right) \quad (5)$$

where T is the number of data points, and Δ^2 is the sum of the squared Mahalanobis distances.

The signal-to-noise ratio and the coupling parameter:
Now we derive the full conditional distributions of λ_u^{-1} and λ_c^{-1}. From the factorized joint density we get:

$$\begin{aligned} p(\lambda_u^{-1}|\ldots) &\propto p(\lambda_u^{-1}) \cdot p(\boldsymbol{\beta}_1|\sigma^2, \lambda_u, \boldsymbol{\pi}, \boldsymbol{\tau}) \cdot \prod_{h=2}^{H} p(\boldsymbol{\beta}_h|\sigma^2, \lambda_u, \lambda_c, \boldsymbol{\delta}, \tilde{\boldsymbol{\beta}}_{h-1}, \boldsymbol{\pi}, \boldsymbol{\tau}) \\ p(\lambda_c^{-1}|\ldots) &\propto p(\lambda_c^{-1}) \cdot \prod_{h=2}^{H} p(\boldsymbol{\beta}_h|\sigma^2, \lambda_u, \lambda_c, \boldsymbol{\delta}, \tilde{\boldsymbol{\beta}}_{h-1}, \boldsymbol{\pi}, \boldsymbol{\tau}) \end{aligned}$$

Next we recall Equations (1-3), and we plug-in the prior Gamma densities of λ_u^{-1} and λ_c^{-1}:

$$\begin{aligned} p(\lambda_u^{-1}|\ldots) &\propto (\lambda_u^{-1})^{a_u-1}\exp\{-b_u\lambda_u^{-1}\}\cdot(\lambda_u^{-1})^{\frac{1}{2}(k+1)}\exp\{-\lambda_u^{-1}\cdot(\frac{1}{2}\sigma^{-2}\cdot\sum_{i=0}^{k}\beta_{1,i}^2)\} \\ &\quad \cdot\prod_{h=2}^{H}(\lambda_u^{-1})^{\frac{1}{2}\sum_{i=0}^{k}(1-\delta_i)}\exp\{-\lambda_u^{-1}\cdot(\frac{1}{2}\sigma^{-2}\cdot\sum_{i:\delta_i=0}\beta_{h,i}^2)\} \\ &\propto (\lambda_u^{-1})^{a_u+\frac{1}{2}k_u-1}\cdot\exp\{-\lambda_u^{-1}(b_u+\frac{1}{2}\sigma^{-2}D_u^2)\} \end{aligned} \quad (6)$$

where $D_u^2 := \sum\limits_{i=0}^{k}\boldsymbol{\beta}_{1,i}^2 + \sum\limits_{h=2}^{H}\sum\limits_{i:\delta_i=0}\boldsymbol{\beta}_{h,i}^2$ and $k_u := (k+1)+(H-1)\cdot\sum_{i=0}^{k}(1-\delta_i)$ is the number of uncoupled regression coefficients.

$$\begin{aligned} p(\lambda_c^{-1}|\ldots) &\propto (\lambda_c^{-1})^{a_c-1}\exp\{-b_c\lambda_c^{-1}\}\cdot \\ &\quad \prod_{h=2}^{H}(\lambda_c^{-1})^{\frac{1}{2}\sum_{i=0}^{k}\delta_i}\exp\{-\lambda_c^{-1}(\frac{1}{2}\sigma^{-2}\cdot\sum_{i:\delta_i=1}(\beta_{h,i}-\tilde{\beta}_{h,i})^2)\} \\ &\propto (\lambda_c^{-1})^{a_c+\frac{k_c}{2}-1}\exp\{-\lambda_c^{-1}(b_c+\frac{1}{2}\sigma^{-2}D_c^2)\} \end{aligned} \quad (7)$$

where $D_c^2 := \sum\limits_{h=2}^{H}\sum\limits_{i:\delta_i=1}(\boldsymbol{\beta}_{h,i}-\tilde{\boldsymbol{\beta}}_{h-1,i})^2$ and $k_c := (H-1)\cdot\sum_{i=0}^{k}\delta_i$ is the number of coupled regression coefficients.

From the shapes of the full conditional densities in Equations (6-7) it follows:

$$FCD(\lambda_u^{-1}) := \lambda_u^{-1}|(\mathbf{y}_1,\ldots,\mathbf{y}_H,\boldsymbol{\beta}_1,\ldots,\boldsymbol{\beta}_H,\sigma^2,\boldsymbol{\delta}) \sim GAM\left(a_u+\frac{k_u}{2}, b_u+\frac{1}{2}\sigma^{-2}D_u^2\right) \quad (8)$$

$$FCD(\lambda_c^{-1}) := \lambda_c^{-1}|(\mathbf{y}_1,\ldots,\mathbf{y}_H,\boldsymbol{\beta}_1,\ldots,\boldsymbol{\beta}_H,\sigma^2,\boldsymbol{\delta}) \sim GAM\left(a_c+\frac{k_c}{2}, b_c+\frac{1}{2}\sigma^{-2}D_c^2\right) \quad (9)$$

Marginal Likelihood:
For the marginal likelihood, with $\boldsymbol{\beta}_h$ $(h=1,\ldots,H)$ and σ^2 integrated out, we apply the rule from Section 2.3.7 of Bishop (2006):

$$p(\mathbf{y}_1,\ldots,\mathbf{y}_H|\lambda_u,\lambda_c,\boldsymbol{\delta}) = \frac{\Gamma(\frac{T}{2}+a_\sigma)}{\Gamma(a_\sigma)}\cdot\frac{\pi^{-T/2}\cdot(2b_\sigma)^{a_\sigma}}{\left(\prod\limits_{h=1}^{H}\det(\mathbf{I}+\mathbf{X}_h\boldsymbol{\Sigma}_h\mathbf{X}_h^{\mathsf{T}})\right)^{1/2}}\cdot(2b_\sigma+\Delta^2)^{-(\frac{T}{2}+a_\sigma)} \quad (10)$$

where Δ^2 and $\boldsymbol{\Sigma}_h$ $(h=1,\ldots,H)$ were defined above.

The vector of indicator variables:

Finally, we derive the full conditional distributions of the elements of the vector $\boldsymbol{\delta} = (\delta_0, \dots, \delta_k)$. As $\delta_0, \dots, \delta_k$ are i.i.d. $BER(p)$ distributed, we get from the factorized joint density:

$$FCD(\delta_i) := p(\delta_i | \dots) \propto p(\mathbf{y}_1, \dots, \mathbf{y}_H | \lambda_u, \lambda_c, \boldsymbol{\delta}) \cdot p(\boldsymbol{\delta}) = p(\mathbf{y}_1, \dots, \mathbf{y}_H | \lambda_u, \lambda_c, \boldsymbol{\delta}) \cdot p^{\delta_i} \cdot (1-p)^{1-\delta_i} \tag{11}$$

And since δ_i is binary, the full conditional is also a Bernoulli distribution:

$$\delta_i | (\lambda_u, \lambda_c, \{\delta_j : j \neq i\}, \boldsymbol{\pi}, \boldsymbol{\tau}, \mathbf{y}) \sim BER(\theta_i)$$

where

$$\theta_i = \frac{p(\delta_i = 1 | \dots)}{p(\delta_i = 1 | \dots) + p(\delta_i = 0 | \dots)} = \frac{p(\mathbf{y} | \lambda_u, \lambda_c, \boldsymbol{\delta}^{\delta_i \leftarrow 1}, \boldsymbol{\pi}, \boldsymbol{\tau}) \cdot p}{\sum_{j=0}^{1} p(\mathbf{y} | \lambda_u, \lambda_c, \boldsymbol{\delta}^{\delta_i \leftarrow j}, \boldsymbol{\pi}, \boldsymbol{\tau}) \cdot p^j \cdot (1-p)^{1-j}}$$

and $\boldsymbol{\delta}^{\delta_i \leftarrow j}$ denotes the vector $\boldsymbol{\delta}$ with δ_i being set to $j \in \{0, 1\}$.

Part B - Metropolis-Hastings sampling (see Section 2.3 of the main paper)

For the posterior density of the new model we have:

$$p(\lambda_u, \lambda_c, \boldsymbol{\delta}, \boldsymbol{\pi}, \boldsymbol{\tau}|\mathcal{D}) \propto p(\mathbf{y}_{\boldsymbol{\mathcal{T}}}|\lambda_u, \lambda_c, \boldsymbol{\delta}, \boldsymbol{\pi}, \boldsymbol{\tau}) \cdot p(\boldsymbol{\pi}) \cdot p(\boldsymbol{\tau}|H) \cdot p(H) \cdot p(\boldsymbol{\delta}) \cdot p(\lambda_u) \cdot p(\lambda_c) \tag{12}$$

where the changepoint set $\boldsymbol{\tau}$ yields the segmentation of the data into H segments, and $\boldsymbol{\pi}$ is the covariate set. The covariates in $\boldsymbol{\pi}$ are used to build the segment-specific design matrices $\mathbf{X}_h$ in the marginal likelihood. If $\boldsymbol{\tau}$ implies the segmentation $\mathbf{y}_{\boldsymbol{\mathcal{T}}} := \{\mathbf{y}_1, \ldots, \mathbf{y}_H\}$, we get:

$$p(\mathbf{y}_{\boldsymbol{\mathcal{T}}}|\lambda_u, \lambda_c, \boldsymbol{\delta}, \boldsymbol{\pi}, \boldsymbol{\tau}) = p(\mathbf{y}_1, \ldots, \mathbf{y}_H|\lambda_u, \lambda_c, \boldsymbol{\delta})$$

with the right-hand side being the marginal likelihood, defined in Equation (10), and the design matrices $\mathbf{X}_1, \ldots, \mathbf{X}_H$ in Equation (10) depending on the covariate set $\boldsymbol{\pi}$.

Given $\boldsymbol{\pi}$ and $\boldsymbol{\tau}$, the parameters λ_u, λ_c and the elements of $\boldsymbol{\delta}$ can be re-sampled from their full conditional distributions, as derived in part A of this supplementary material and summarized in Section 2.2 of the main paper.[1] Given λ_u, λ_c, and $\boldsymbol{\delta}$, Metropolis-Hastings steps can be used to sample the covariate set $\boldsymbol{\pi}$ and the changepoint set $\boldsymbol{\tau}$.

Moves on the covariate set: For sampling $\boldsymbol{\pi}$ from the posterior we implement 3 moves:

- **Covariate Removal (R)**: We randomly select one covariate $X_i \in \boldsymbol{\pi}$ and remove it from $\boldsymbol{\pi}$. Along with the covariate we also delete the corresponding element δ_i of $\boldsymbol{\delta}$.
- **Covariate Addition (A)**: We randomly select one covariate $X_i \notin \boldsymbol{\pi}$ and add it to $\boldsymbol{\pi}$. Along with the new covariate we also add a new element δ_i to $\boldsymbol{\delta}$. We flip a coin to determine the value of δ_i.
- **Covariate Exchange (E)**: We randomly select one covariate $X_i \in \boldsymbol{\pi}$ and we replace it by a randomly selected new covarite$X_j \notin \boldsymbol{\pi}$. We remove δ_i from $\boldsymbol{\delta}$ and add δ_j to $\boldsymbol{\delta}$. We flip a coin to determine the value of δ_j.

Each move proposes to replace $[\boldsymbol{\pi}, \boldsymbol{\delta}]$ by $[\boldsymbol{\pi}^*, \boldsymbol{\delta}^*]$. When randomly selecting the move type, the acceptance probabilities are:

$$A([\boldsymbol{\pi}, \boldsymbol{\delta}] \to [\boldsymbol{\pi}^*, \boldsymbol{\delta}^*]) = \min\left\{1, \frac{p(\mathbf{y}|\lambda_u, \lambda_c, \boldsymbol{\delta}^*, \boldsymbol{\pi}^*, \boldsymbol{\tau})}{p(\mathbf{y}|\lambda_u, \lambda_c, \boldsymbol{\delta}, \boldsymbol{\pi}, \boldsymbol{\tau})} \cdot \frac{p(\boldsymbol{\pi}^*)}{p(\boldsymbol{\pi})} \cdot \frac{p(\boldsymbol{\delta}^*)}{p(\boldsymbol{\delta})} \cdot HR\right\} \tag{13}$$

where the Hastings Ratio HR depends on the move type:

$$HR_R = \frac{|\boldsymbol{\pi}|}{n - |\boldsymbol{\pi}^*|} \cdot 0.5, \qquad HR_A = \frac{n - |\boldsymbol{\pi}|}{|\boldsymbol{\pi}^*|} \cdot 2, \qquad HR_E = 1$$

where n is the number of potential covariates, $|.|$ denotes the cardinality, and the factors 2 and 0.5 stem from flipping coins for the values of newly introduced indicator variables.[2]

[1] The parameters σ^2 and $\boldsymbol{\beta}_1, \ldots, \boldsymbol{\beta}_H$ are marginalized out in Equations (10) and (12). But they have to be sampled, before sampling from the full conditionals of λ_u and λ_c in Equations (8-9).

[2] For $p = 0.5$ the prior ratio $p(\boldsymbol{\delta}^\star)/p(\boldsymbol{\delta})$ cancels with the factors 2 and 0.5, respectively.

Moves on the changepoint set: For sampling $\boldsymbol{\tau}$ we also implement 3 moves:

- **Changepoint Birth (B)**: Out of the set of all valid new changepoint locations $\mathcal{B}(\boldsymbol{\tau})$ we randomly sample one element and propose to set a new changepoint at this location. The new changepoint set $\boldsymbol{\tau}^*$ contains $H^* = H + 1$ changepoints.
- **Changepoint Death (D)**: We randomly select one changepoint $\tau \in \boldsymbol{\tau}$ and delete it. The new changepoint set $\boldsymbol{\tau}^*$ contains $H^* = H - 1$ changepoints.
- **Changepoint Reallocation (R)**: We randomly select one changepoint $\tau_j \in \boldsymbol{\tau}$ and propose to re-allocate it to a randomly selected position in between the two surrounding changepoints. The valid positions are: $\tau_{j-1} + 2, \ldots, \tau_{j+1} - 2$. The changepoint reallocation yields the new changepoint set $\boldsymbol{\tau}^*$ with $H^* = H$ changepoints.

When randomly selecting the move type, the acceptance probabilities are:

$$A([\boldsymbol{\tau}, H] \to [\boldsymbol{\tau}^*, H^*]) = \min\left\{1, \frac{p(\mathbf{y}_{\boldsymbol{\tau}^*}|\lambda_u, \lambda_c, \boldsymbol{\delta}, \boldsymbol{\pi}, \boldsymbol{\tau}^*)}{p(\mathbf{y}_{\boldsymbol{\tau}}|\lambda_u, \lambda_c, \boldsymbol{\delta}, \boldsymbol{\pi}, \boldsymbol{\tau})} \cdot \frac{p(\boldsymbol{\tau}^*|H^*)}{p(\boldsymbol{\tau}|H)} \cdot \frac{p(H^*)}{p(H)} \cdot HR\right\} \tag{14}$$

where the Hastings Ratio HR depends on the move type:

$$HR_B = \frac{|\mathcal{B}(\boldsymbol{\tau})|}{|\boldsymbol{\tau}^*|}, \qquad HR_D = \frac{|\boldsymbol{\tau}|}{|\mathcal{B}(\boldsymbol{\tau}^*)|}, \qquad HR_R = 1$$

where $\mathcal{B}(\boldsymbol{\tau}) := \{\tau | 2 \leq \tau \leq T-1 \ \textbf{ and } \ |\tau_j - \tau| \geq 2 \ \text{ for } j = 1, \ldots, H-1\}$ is the set of all valid new changepoint locations, given $\boldsymbol{\tau} = \{\tau_1, \ldots, \tau_H\}$, and $|.|$ denotes the cardinality.

Pseudo code for RJMCMC sampling algorithm

Given the data, $\mathcal{D}$ and one single target node, we use Reversible Jump Markov Chain Monte Carlo (RJMCMC) simulations to generate a sample $\{\boldsymbol{\pi}^{(w)}, \boldsymbol{\tau}^{(w)}, \lambda_u^{(w)}, \lambda_c^{(w)}, \boldsymbol{\delta}^{(w)}\}_{w=1,\ldots,W}$ from the posterior distribution $p(\boldsymbol{\pi}, \boldsymbol{\tau}, \lambda_u, \lambda_c, \boldsymbol{\delta}|\mathcal{D})$. In each iteration ($w = 1, \ldots, W$) of the RJMCMC algorithm we first re-sample the parameters σ^2, $\boldsymbol{\beta}_1, \ldots, \boldsymbol{\beta}_H$, λ_u, λ_c and $\boldsymbol{\delta}$ from their full conditional distributions (Gibbs sampling), before we perform two Metropolis-Hastings moves; one on the covariate set $\boldsymbol{\pi}$ and one on the changepoint set $\boldsymbol{\tau}$. Table 1 gives pseudo code for one single iteration ($w \to w+1$) of the RJMCMC algorithm for generating the sample $\{\boldsymbol{\pi}^{(w)}, \boldsymbol{\tau}^{(w)}, \lambda_u^{(w)}, \lambda_c^{(w)}, \boldsymbol{\delta}^{(w)}\}_{w=1,\ldots,W}$.

For inferring the network structure for a domain with N nodes, we have to implement a nested loop. An additional 'outer' loop is used to iterate through the individual network variables $Z_1, \ldots, Z_N$. In the i-th iteration of the outer loop ($i = 1, \ldots, N$) we generate a posterior sample $\{\boldsymbol{\pi}_i^{(w)}, \boldsymbol{\tau}_i^{(w)}, \lambda_{u,i}^{(w)}, \lambda_{c,i}^{(w)}, \boldsymbol{\delta}_i^{(w)}\}_{w=1,\ldots,W}$ for response Z_i by iterating through the (inner) loop ($w = 1, \ldots, W$). Subsequently, as explained in Sections 2.4 and 2.5 of the main paper, we can merge the sampled covariate sets to a network sample $\{\mathcal{G}^{(w)}\}_{w=1,\ldots,W}$, where $\mathcal{G}^{(w)} := (\boldsymbol{\pi}_1^{(w)}, \ldots, \boldsymbol{\pi}_N^{(w)})$.

Input: The data $\mathcal{D}$ and the current instantiations of the covariate set $\boldsymbol{\pi}^{(w)}$, the changepoint set $\boldsymbol{\tau}^{(w)}$, the vector $\boldsymbol{\delta}^{(w)}$, and the parameters $\lambda_u^{(w)}$ and $\lambda_c^{(w)}$.

RJMCMC iteration: $w \to w+1$:

- Sample a new noise variance parameter $\sigma_\diamond^{-2}$ from
  $\sigma^{-2}|(\mathbf{y}_1, \ldots, \mathbf{y}_H, \lambda_u^{(w)}, \lambda_c^{(w)}, \boldsymbol{\delta}^{(w)}, \boldsymbol{\pi}^{(w)}, \boldsymbol{\tau}^{(w)})$, see Equation (5).

- For $h = 1, \ldots, H$
  - Sample the segment-specific regression coefficients vector $\boldsymbol{\beta}_h^\diamond$ from
    $\boldsymbol{\beta}_h|(\mathbf{y}_h, \sigma_\diamond^2, \lambda_u^{(w)}, \lambda_c^{(w)}, \boldsymbol{\delta}^{(w)}, \boldsymbol{\pi}^{(w)}, \boldsymbol{\tau}^{(w)})$, see Equation (4).

- Sample $\lambda_{u,\diamond}^{-1}$ from $\lambda_u^{-1}|(\boldsymbol{\beta}_1^\diamond, \ldots, \boldsymbol{\beta}_H^\diamond, \sigma_\diamond^2, \boldsymbol{\delta}^{(w)}, \boldsymbol{\pi}^{(w)}, \boldsymbol{\tau}^{(w)})$, see Equation (8).
  Invert the sampled value $\lambda_{u,\diamond}^{-1}$ to obtain $\lambda_u^{(w+1)}$

- Sample $\lambda_{c,\diamond}^{-1}$ from $\lambda_c^{-1}|(\boldsymbol{\beta}_1^\diamond, \ldots, \boldsymbol{\beta}_H^\diamond, \sigma_\diamond^2, \boldsymbol{\delta}^{(w)}, \boldsymbol{\pi}^{(w)}, \boldsymbol{\tau}^{(w)})$, see Equation (9).
  Invert the sampled value $\lambda_{c,\diamond}^{-1}$ to obtain: $\lambda_c^{(w+1)}$

- Withdraw $\boldsymbol{\beta}_1^\diamond, \ldots, \boldsymbol{\beta}_H^\diamond$, $\sigma_\diamond^2$, $\lambda_{u,\diamond}^{-1}$ and $\lambda_{c,\diamond}^{-1}$. That is, keep only $\lambda_u^{(w+1)}$ and $\lambda_c^{(w+1)}$.

- Randomly select one of the $k+1$ elements of the vector $\boldsymbol{\delta}^{(w)}$. Replace the selected element $\delta_i^{(w)}$ by a new value $\delta_i^{(w+1)}$ where the latter is sampled from
  $\delta_i|(\lambda_u^{(w+1)}, \lambda_c^{(w+1)}, \{\delta_j^{(w)} : j \neq i\}, \boldsymbol{\pi}^{(w)}, \boldsymbol{\tau}^{(w)}, \mathbf{y})$, see Equation (11).
  Replacing the element $\delta_i^{(w)}$ of $\boldsymbol{\delta}^{(w)}$ by $\delta_i^{(w+1)}$ yields the new vector $\boldsymbol{\delta}^{(w+1)}$.

- Metropolis-Hastings move on the covariate set $\boldsymbol{\pi}^{(w)}$:
  - Randomly select the move type (R, A or E), and propose to move from $[\boldsymbol{\pi}^{(w)}, \delta^{(w)}]$ to $[\boldsymbol{\pi}^*, \delta^*]$. Accept the new state $[\boldsymbol{\pi}^*, \delta^*]$ with the acceptance probability given in Equation (13) with $\lambda_u = \lambda_u^{(w+1)}$, $\lambda_c = \lambda_c^{(w+1)}$, $\boldsymbol{\delta} = \boldsymbol{\delta}^{(w+1)}$, $\boldsymbol{\pi} = \boldsymbol{\pi}^{(w)}$, $\boldsymbol{\delta} = \boldsymbol{\delta}^{(w)}$.
  - If the move is accepted, set: $\boldsymbol{\pi}^{(w+1)} = \boldsymbol{\pi}^*$ and $\boldsymbol{\delta}^{(w+1)} = \boldsymbol{\delta}^*$.
    Otherwise set: $\boldsymbol{\pi}^{(w+1)} = \boldsymbol{\pi}^{(w)}$ and $\boldsymbol{\delta}^{(w+1)} = \boldsymbol{\delta}^{(w)}$.

- Metropolis-Hastings move on the changepoint set $\boldsymbol{\tau}^{(w)}$:
  - Randomly select the move type (B, D or R), and propose to move from $[\boldsymbol{\tau}^{(w)}, H^{(w)}]$ to $[\boldsymbol{\tau}^*, H^*]$. Accept the new state $[\boldsymbol{\tau}^*, H^*]$ with the acceptance probability given in Equation (14) using $\lambda_u = \lambda_u^{(w+1)}$, $\lambda_c = \lambda_c^{(w+1)}$, $\boldsymbol{\delta} = \boldsymbol{\delta}^{(w+1)}$, $\boldsymbol{\pi} = \boldsymbol{\pi}^{(w+1)}$, $\boldsymbol{\tau} = \boldsymbol{\tau}^{(w)}$.
  - If the move is accepted, set: $\boldsymbol{\tau}^{(w+1)} = \boldsymbol{\tau}^*$ and $H^{(w+1)} = H^*$.
    Otherwise set: $\boldsymbol{\tau}^{(w+1)} = \boldsymbol{\tau}^{(w)}$ and $H^{(w+1)} = H^{(w)}$.

Output: The re-sampled instantiations: $\boldsymbol{\pi}^{(w+1)}$, $\boldsymbol{\tau}^{(w+1)}$, $\boldsymbol{\delta}^{(w+1)}$, $\lambda_u^{(w+1)}$, and $\lambda_c^{(w+1)}$.

Table 1: **Pseudo code.** The table summarizes one iteration ($w \to w+1$) of the RJMCMC algorithm for one single regression model with response Y and the potential covariates $X_1, \ldots, X_n$.

Part C - Extended texts

C.1 Potential Scale Reduction Factors (Section 3.1 of the main paper)

The RJMCMC convergence can be monitored in terms of PSRFs; see, e.g. Brooks and Gelman (1998). We perform H independent RJMCMC simulations and for each simulation h we compute the score $\hat{e}_{i,j}^{(h,s)}$ of edge $Z_i \to Z_j$ after $200s$ ($s = 1, \ldots, 500$) iterations. Assuming a burn-in of $100s$ iterations and thinning out by the factor 100, yields s samples and we compute the "between-chain" and the "within-chain" variances:

$$\mathcal{B}_s(i,j) = \frac{1}{H-1}\sum_{h=1}^{H}(\hat{e}_{i,j}^{(h,s)} - \bar{e}_{i,j}^{(.,s)})^2 \text{ and } \mathcal{W}_s(i,j) = \frac{1}{H(s-1)}\sum_{h=1}^{H}\sum_{w=1}^{s}(I_{i\to j}(\mathcal{G}_h^{(w)}) - \hat{e}_{i,j}^{(h,s)})^2$$

where $\bar{e}_{i,j}^{[.,s]}$ is the mean of $\hat{e}_{i,j}^{(1,s)}, \ldots, \hat{e}_{i,j}^{(H,s)}$, and $I_{i\to j}(\mathcal{G}_h^{(w)})$ is 1 if network w of simulation h has the edge $Z_i \to Z_j$, and 0 otherwise. After $200s$ iterations the PSRF of the edge $Z_i \to Z_j$ is:

$$PSRF_s(i,j) = \frac{(1-\frac{1}{s})\mathcal{W}_s(i,j) + (1+\frac{1}{H})\mathcal{B}_s(i,j)}{\mathcal{W}_s(i,j)} \tag{15}$$

PSRFs near 1 indicate that the RJMCMC simulations are close to the stationary distribution. We monitor the fraction of edges with $PSRF < 1.01$ against the RJMCMC iterations $200s$.

For all data sets all PSRF's were below 1.01 after 100k iterations. Figure 1 shows the convergence monitors for the yeast data and for the Arabidopsis data.

C.2 Yeast gene expression data (Section 4.2 of the main paper)

By means of synthetic biology Cantone *et al.* (2009) designed a network with $N = 5$ genes and $M = 8$ edges in *S. cerevisiae* (yeast). With quantitative Real-Time Polymerase Chain Reaction (RT-PCR), Cantone *et al.* (2009) then measured in vivo gene expression data: first under galactose- and then under glucose-metabolism. For both carbon sources the network structure is identical, but the strengths of the regulatory processes (i.e. the network parameters) change with the carbon source (Cantone *et al.* (2009)). For each gene Z_i, 16 measurements were taken in galactose $d_1^i, \ldots, d_{16}^i$ and 21 measurements were taken in glucose $d_1^{i,*}, \ldots, d_{21}^{i,*}$, with 20 minutes intervals in between measurements. For both parts of the time series the initial measurements d_1^i and $d_1^{i,*}$ were taken while extant glucose (galactose) was washed out and new galactose (glucose) was supplemented. We withdraw the initial measurements from the washing period, before we re-merge the two time series parts. After a gene-wise zscore-standardization (to mean 0 and variance 1) we build for each gene Z_i the response vector $\mathbf{y} = (d_3^i, \ldots, d_{16}^i, d_3^{i,*}, \ldots, d_{21}^{i,*})^\mathsf{T}$ and use the other genes Z_j $(j \neq i)$ as covariates. For explaining $\mathbf{y}$, we use the shifted values: $(d_2^j, \ldots, d_{15}^j, d_2^{j,*}, \ldots, d_{20}^{j,*})^\mathsf{T}$.

C.3 Arabidopsis gene expression data (Section 4.3 of the main paper)

The circadian clock in *Arabidopsis thaliana* synchronizes the plant metabolism with the daily 24-h photo period (i.e. with the daily dark:light cycle), which is caused by the rotation of the earth. The circadian clock is capable of anticipating the external cycle and can thus optimize the gene regulatory processes w.r.t. the expected (=entrained) photo period. Thereby the structure

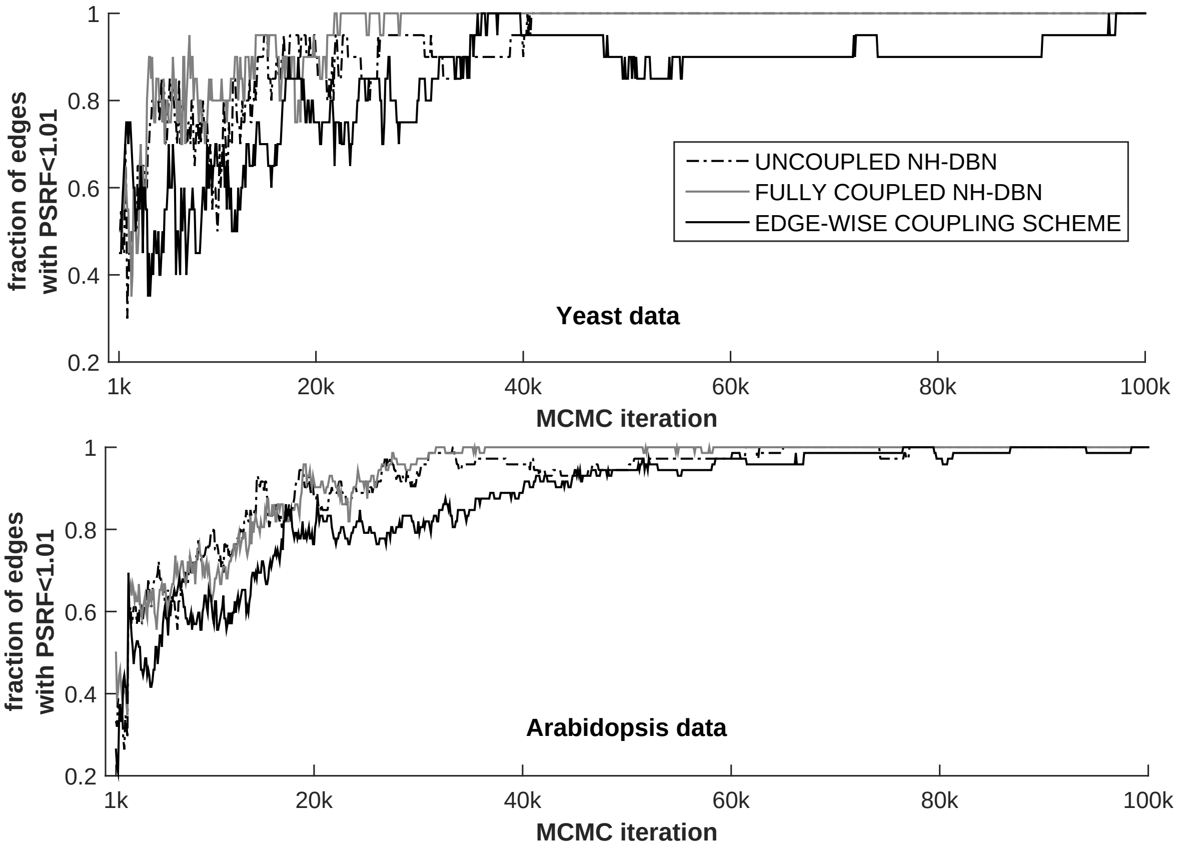

Figure 1: **Convergence diagnostics based on potential scale reduction factors (PSRFs).** For the yeast and the Arabidopsis gene expression data, we ran $H = 10$ RJMCMC simulations and for each edge we computed a PSRF. The plots show the fractions of edges that fulfilled: $PSRF < 1.01$, monitored along the RJMCMC iterations.

of the regulatory network does not change, but the strengths of the gene interactions depend on the entrained photo period. In four experiments (E1-E4) Arabidopsis plants were entrained in different dark:light cycles, before data were collected every 2 or 4 hours under constant light:

- **E1**: Dark:light entrainment: **12h:12h**, then 12 measurements at **4h** intervals.
- **E2**: Dark:light entrainment: **12h:12h**, then 13 measurements at **4h** intervals.
- **E3**: Dark:light entrainment: **10h:10h**, then 13 measurements at **2h** intervals.
- **E4**: Dark:light entrainment: **14h:14h**, then 13 measurements at **2h** intervals.

RNA was measured using Affymetrix microarrays and an RMA normalization was applied. We concentrate on the $N = 9$ core clock genes: LHY, TOC1, CCA1, ELF4, ELF3, GI, PRR9, PRR5, and PRR3, and we merge the data into one single time series by arranging the individual data successively. For each of the four initial points we do not have values for the potential covariates, so that we cannot use them as response values.
For the four individual time series and more detailed information about the experimental settings and entrainments, we refer to Mockler *et al.* (2007) (**E1**), Edwards *et al.* (2006) (**E2**) and Grzegorczyk *et al.* (2008) (**E3** and **E4**).

Part D - Network reconstruction accuracy when self-loops are valid (see Section 3 of the main paper)

In this section we provide empirical evidence that the allowance of self-loops (autoregressive edges), like $Z_i \to Z_i$, can have negative effects on the network reconstruction accuracy. We therefore allowed for self (feedback) loops and re-analysed the yeast data with all NH-DBN models from Section 2.6 of the main paper. The two panels of Figure 2 show histograms of the AUC results when self-loops are ruled out (left panel) and when self-loops are considered to be valid edges (right panel). When comparing the two histograms it becomes obvious that the NH-DBN models reach substantially lower average AUC values when self-loops are allowed. The proposed EWC NH-DBN yields the highest AUC score in both settings. We note that we currently do not have a proper theoretical explanation why allowing for self-loops diminishes the network reconstruction accuracy of NH-DBN models. In our future work we will investigate this phenomenon in more detail and elaborate the reason(s).

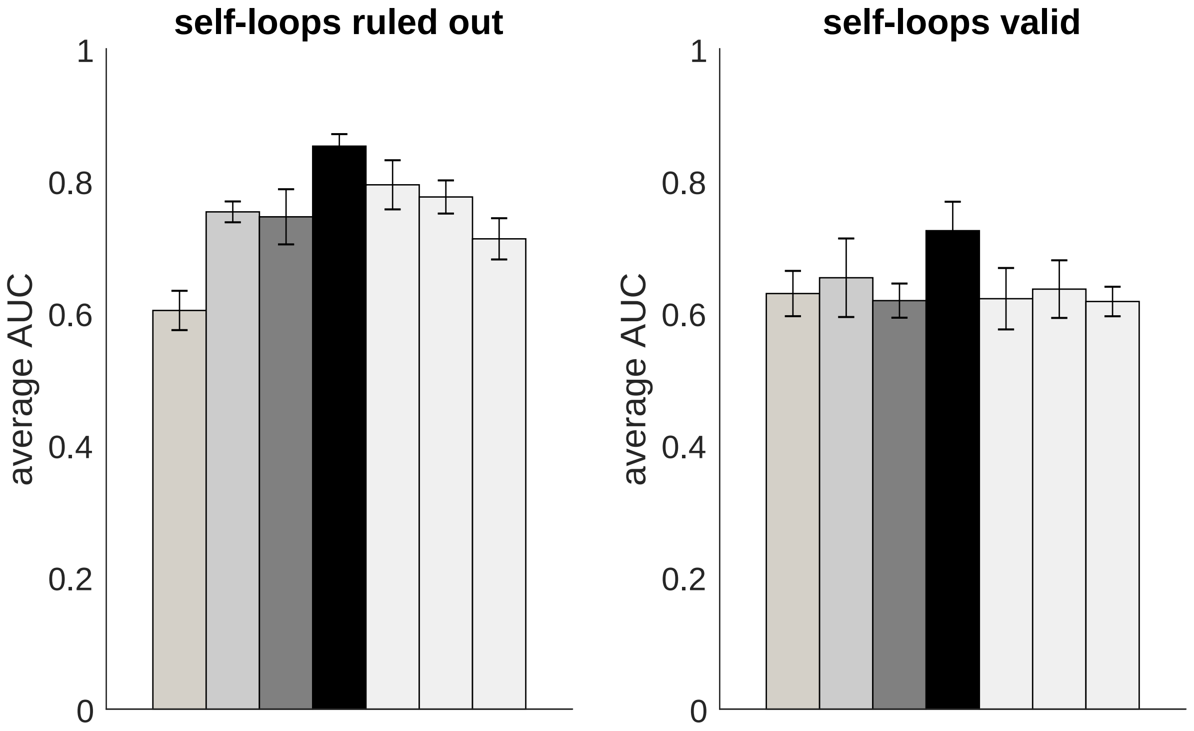

Figure 2: **Comparison of the network reconstruction accuracies under two different settings: without self-loops (left histogram) and with self-loops (right histogram).** In both histograms the bars (from left to right) refer to the models: M1, M2, M3, EWC, M6, M5, and M4. The left histogram shows the average precision-recall AUC values when auto-regressive self-loop edges are ruled out (i.e. when they cannot be inferred). The left histogram is thus a reproduction of the left histogram in Figure 6 of the main paper. The right histogram is new and shows the average precision-recall AUC values when self-loops are considered to be valid edges (i.e. when they can be inferred). It can be clearly seen that the AUCs in the right histogram are lower; except for the DBN (M1). In both settings the proposed EWC NH-DBN reaches the highest network reconstruction accuracy (black bars of the two histograms).

Part E - Computational costs (see Section 3 of the main paper)

In this section we study how the new EWC NH-DBN scales up to larger networks.
We re-use the synthetic data generation mechanism from Section 4.1 of the main paper. But instead of generating data from the RAF network with $N = 11$ nodes, we now generate data for random networks with $N = 10, 25, 50, 100$ nodes. For every node Z_i ($i = 1, \dots, N$) we sample the cardinality p_i ($= |\boldsymbol{\pi}_i|$) of its covariate set $\boldsymbol{\pi}_i$ from a Poisson distribution with parameter $\lambda = 1$. This yields a network structure with so called 'Poisson in-degree distribution'. Subsequently we randomly draw the parent set $\boldsymbol{\pi}_i$ from a uniform distribution over the system of all possible parent sets with cardinality p_i, i.e. from a uniform distribution over the system $\{\boldsymbol{\pi}_i : |\boldsymbol{\pi}_i| = p_i\}$. Having sampled a parent set for each node, we generate data sets as described in Section 4.1 of the main paper. That is, we use the same regression model equation (see Section 4.1 of the main paper) and we just exchange the RAF network structure by random network structures. We keep the number of segments fixed at $H = 4$, but we vary the number of observations per segment $m = 10, 25, 50, 100$.[3] We here report the results for the scenario 'mixture of T1&T4' with 50% of the edges being coupled (T1).[4] For each of the 16 combinations of N and m ($m, N \in \{10, 25, 50, 100\}$), we generate 5 independent data sets (80 data sets in total), and we run two independent RJMCMC simulations on each data set (160 simulations in total).
First we measure the average computational costs for 10,000 RJMCMC iterations. To this end, we run our Matlab RJMCMC code on a computer cluster, whose individual nodes have an Intel Xeon 2.5 GHz processor with 8GB of RAM. The average computational costs (per network and per node) are listed in Tables 2-3. From those measurements it can be easily computed how many RJMCMC iterations can be run per hour; see Table 4.
After this pre-analysis of the computational costs, we start the 160 individual RJMCMC simulations using the numbers of RJMCMC iterations from Table 4. During each RJMCMC simulation we sample 200 equidistant network structures, from which we later withdraw the first 100 networks to take the 'burn-in phase' into account. To assess convergence, we consider scatter plots of the edge scores of two independent RJMCMC simulations on the same data set. For each combination of N and m there is a panel in Figure 3 showing 5 superimposed scatter plots. The scatter plots indicate good convergence for the smaller networks ($N = 10$ and $N = 25$), while the rate of convergence for the larger networks ($N = 50$ and especially $N = 100$) is not satisfactory yet. To get an impression of the network reconstruction accuracies, we average for each data set the edge scores of the two independent RJMCMC simulations and compute the precision-recall AUCs from the average scores; see Table 5. The trends meet our expectations: (i) The AUCs seem to increase with the data set size m, and (ii) the AUCs appear to decrease with the network size N.
For the largest networks ($N = 100$) we run longer RJMCMC simulations, taking 12 hours of computational time. Figure 4 shows the scatter plots after 1.5, 3, 6 and 12 hours of computational time. After 6-12 hours only very few points deviate from the diagonal, i.e. only few edge scores differ between independent simulations. We would thus argue that even networks with $N = 100$ can be inferred in a reasonable time: On a desktop computer 6-12 hours have to be invested to reach sufficient convergence. When a computer cluster is available, the network inference task can be subdivided into N independent covariate set inference tasks and each of the $N = 100$ individual simulations would take around 3.6 to 7.2 minutes (6-12 hours in total).

[3] We use different values of m, so as to obtain flat (for low m) and peaked (for high m) posterior landscapes.

[4] For the other two scenarios ('mixture of T1&T2' and 'mixture of T1&T3') as well as for other percentages of coupled edges (T1) we observed very similar convergence results.

	**N=10**	**N=25**	**N=50**	**N=100**
**m=10**	3.76	11.55	23.81	49.18
**m=25**	4.71	12.40	26.09	53.57
**m=50**	5.94	14.22	29.41	64.51
**m=100**	7.93	20.91	42.86	88.24

Table 2: **Average computational costs for 10,000 network inference RJMCMC iterations on a computer with Intel Xeon 2.5 GHz processor and 8GB of RAM [in minutes].** Columns refer to the network size (N nodes); rows refer to data set sizes (m observations per segment). For the network inference the RJMCMC algorithm was run N times (i.e. for each node $Z_1, \dots, Z_N$). For the computational costs per individual network node Z_i see Table 3.

	**N=10**	**N=25**	**N=50**	**N=100**
**m=10**	22.6	27.7	28.6	29.5
**m=25**	28.3	29.8	31.3	32.2
**m=50**	35.6	34.1	35.3	38.7
**m=100**	47.6	50.2	51.5	52.9

Table 3: **Computational costs for 10,000 RJMCMC iterations per individual network node $\mathbf{Z_i}$ [in seconds].**

	**N=10**	**N=25**	**N=50**	**N=100**
**m=10**	159.3	52.0	25.2	12.2
**m=25**	127.4	48.4	23.0	11.2
**m=50**	101.0	42.2	20.4	9.3
**m=100**	75.7	28.7	14.0	6.8

Table 4: **Average number of network inference RJMCMC iterations per hour [in thousand] on a computer with Intel Xeon 2.5 GHz processor and 8GB of RAM.** In one hour those numbers of RJMCMC iterations can be performed N times (i.e. for all network nodes $Z_1, \dots, Z_N$).

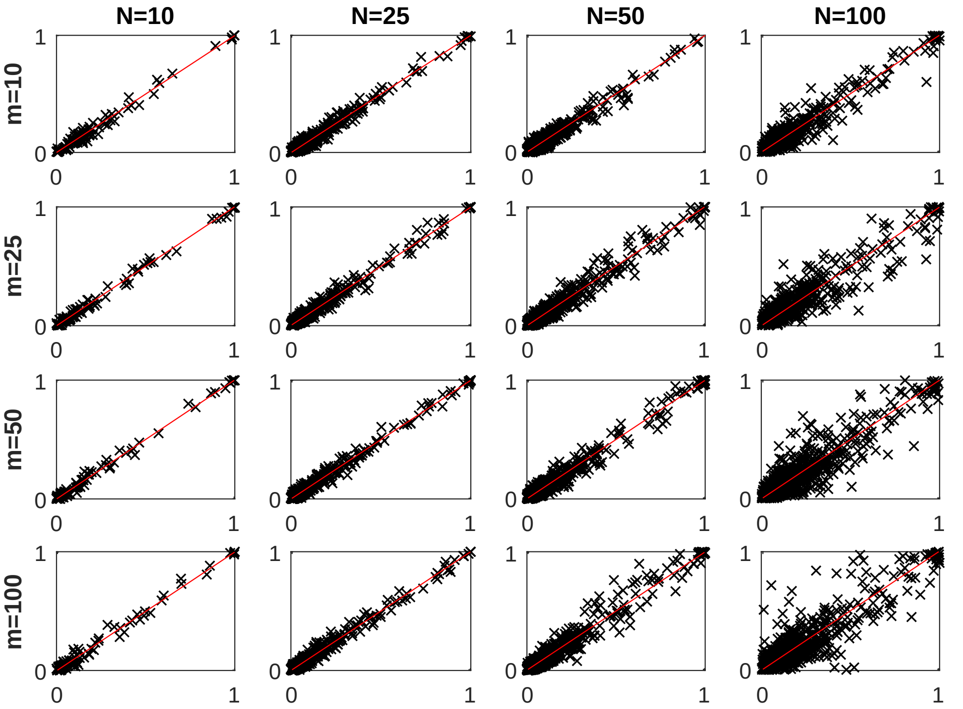

Figure 3: **Convergence diagnostics after one hour of computational time on a computer with Intel Xeon 2.5 GHz processor and 8GB of RAM.** Edge score scatter plots for networks with $N \in \{10, 25, 50, 100\}$ nodes and $m \in \{10, 25, 50, 100\}$ observations per segment. For each combination of N and m, five independent data instantiations were generated, and for each data set, two independent RJMCMC simulations were run. After one hour of computational time, from both RJMCMC outputs the edge scores were computed and plotted against each other. Each panel shows 5 overlaid scatter plots.

	**N=10**	**N=25**	**N=50**	**N=100**
**m=10**	0.56±0.25	0.30±0.13	0.26±0.06	0.19±0.08
**m=25**	0.69±0.20	0.46±0.03	0.31±0.07	0.26±0.08
**m=50**	0.71±0.17	0.46±0.12	0.28±0.08	0.23±0.06
**m=100**	0.74±0.17	0.43±0.15	0.42±0.06	0.34±0.10

Table 5: **Mean AUC values (± standard deviations) after one hour of computational time.** We averaged for each data set the edge scores of the two independent RJMCMC simulations and computed the AUC from the average scores. The table shows the mean and the standard deviation of 5 AUC values.

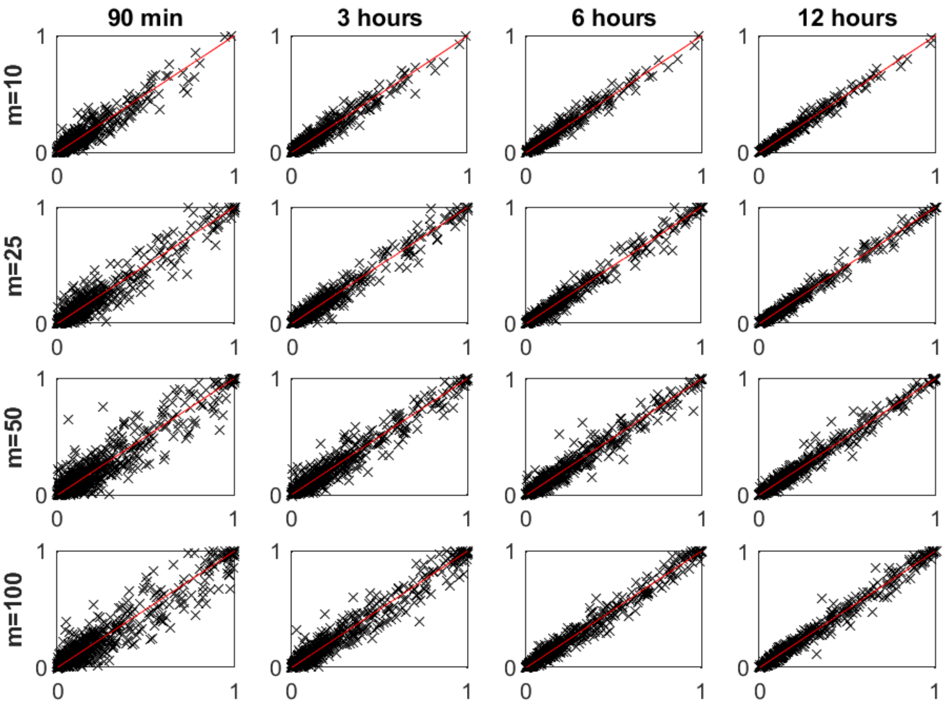

Figure 4: **Convergence diagnostics for large networks.** Edge score scatter plots for the large networks with $N = 100$ nodes and m observations per segment ($m \in \{10, 25, 50, 100\}$). For each m, five independent data instantiations were generated, and for each data set, two independent RJMCMC simulations were run. From both RJMCMC outputs the edge scores were computed after 1.5, 3, 6 and 12 hours of computational time on a computer with Intel Xeon 2.5 GHz processor and 8GB of RAM. The edge scores after 1.5, 3, 6 and 12 hours were then plotted against each other, so as to monitor convergence over time. Each panel shows 5 overlaid scatter plots.

Part F - Additional figure for RAF-pathway data (see Section 5.1 of the main paper)

Figure 5 of this supplementary paper refers to Figure 4 of the main paper and shows the average total AUC scores of the new EWC NH-DBN and the competing methods. The resulting AUC differences have been shown in Figure 4 of the main paper.

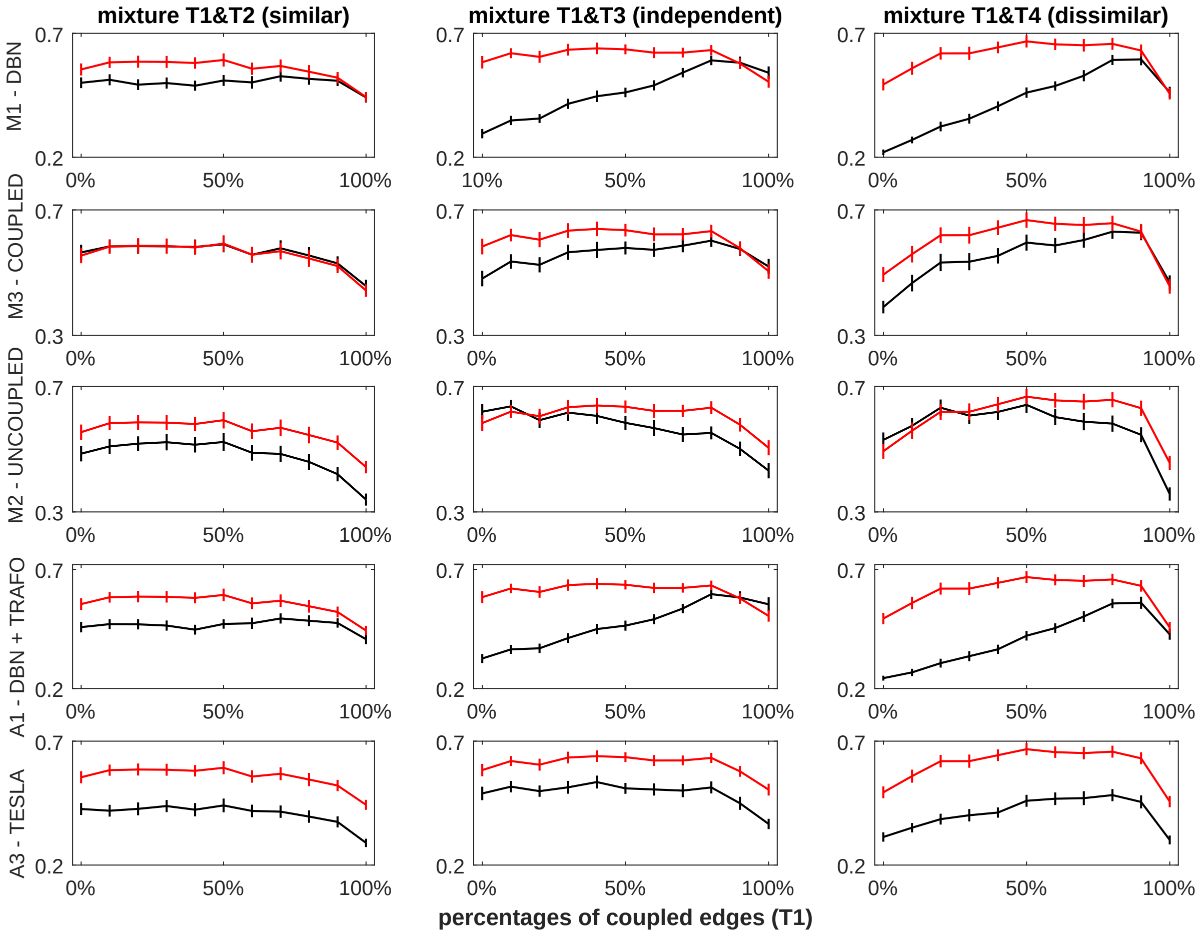

Figure 5: **Total AUC values for the synthetic RAF-pathway data.** This supplementary figure corresponds to Figure 4 of the main paper. Instead of the relative AUC differences, this figure shows the total AUC values. The columns refer to the three scenarios (mixtures of $T1\&T2$, $T1\&T3$, and $T1\&T4$) and the rows refer to the 5 competing methods (M1, M3, M2, A1, and A3). In the panels the AUC values (averaged across 100 data instantiations) have been plotted against the percentage of coupled edges (T1). The black curves refer to the AUCs of the competing methods. The red curves refer to the AUCs of the new EWC NH-DBN. The red curves are thus identical in each column. The error bars on the curves correspond to 0.95 confidence intervals of one-sample t-tests.

Part G - Yeast data (see Section 5.2 of the main paper)

G.1 Relative AUC score differences (supplement for Section 5.2)

In this section we provide overview tables with the relative AUC differences for the yeast data.

**NH-DBN models**	M1	M2	M3	**EWC**	M6	M5	M4
M1: DBN	——						
M2: UNCOUPLED NH-DBN	**0.15**	——	0.00				0.04
M3: COUPLED NH-DBN	**0.14**		——				0.03
**EWC NH-DBN (PROPOSED)**	**0.25**	**0.10**	**0.11**	——	**0.06**	**0.08**	**0.14**
M6: PARTIALLY SEGMENT-WISE	**0.19**	0.04	0.05		——	0.02	**0.08**
M5: SWITCH NH-DBN	**0.17**	0.02	0.03			——	**0.06**
M4: GENERALIZED	**0.11**						——

Table 6: **AUC differences between the EWC NH-DBN and the related NH-DBNs from Section 2.6 of the main paper.** The total AUC values are shown in the left histogram in Figure 6 of the main paper. This table provides the relative differences. We only list the 21 positive differences, and the differences are always in favour of the model indicated in the row. For each comparison we also computed the p-value of a two-sided unpaired two-sample t-test. Significant differences ($p < 0.05$) are in bold. For example, the 1st column shows that the AUCs of the DBN (M1) are significantly lower than the AUCs of any other method. The 4th row shows that the AUCs of the EWC NH-DBN are significantly higher than the AUCs of the six other methods (M1-M6).

**Network models**	A1	A2	A3	A4	**EWC**	A5	A6	A7
A1: DBN + TRAFO	——							
A2: NH-DBN NON-+ TRAFO	**0.13**	——						
A3: TESLA	**0.26**	**0.13**	——			0.01	*0.08*	*0.13*
A4: HMM NH-DBN	**0.28**	**0.15**	0.02	——		0.03	**0.10**	**0.15**
**EWC NH-DBN (PROPOSED)**	**0.33**	**0.20**	**0.07**	**0.05**	——	**0.08**	**0.15**	**0.20**
A5: CHEMA	**0.26**	**0.12**				——	**0.07**	**0.12**
A6: GP4GRN	**0.19**	0.05					——	*0.05*
A7: NeRDS	**0.13**	0.00						——

Table 7: **AUC differences between the EWC NH-DBN and the alternative methods from Section 2.7 of the main paper.** The total AUC values are shown in the right histogram in Figure 7 of the main paper. This table provides the relative differences. We only list the 28 positive differences, and the differences are always in favour of the model in the row. We computed t-test p-values, and the significant differences ($p < 0.05$) are in bold. Three methods (A3, A6 and A7) yield deterministic AUC values. When comparing those three methods with the other methods, we applied one-sample t-tests. For comparisons among A3, A6 and A7 we cannot compute p-values. The corresponding three pairwise differences are *in italics*.

Part G.2 - Changepoint inference (supplement for Section 5.2)

Figure 6 shows the inferred marginal posterior probability of the changepoint locations for the yeast data. From the averaged posterior probabilities in the lower right panel it can be seen that the true changepoint from galactose to glucose is detected. However, the individual posterior probabilities suggest that the $N = 5$ genes are affected by this carbon source switch to different extent. E.g. for gene CBF1 the changepoint is clearly detected, while the peak for gene GAL4 is rather low. Also it can be seen there are additional locations for which high posterior probabilities for a changepoint have been inferred, e.g. at $t = 8$ (CBF1) and $t = 23$ (ASH1). Also it is noteworthy that the posterior probability peak for genes SWI5 and GAL80 appears slightly premature. A possible explanation could be that the galactose medium was already substantially reduced towards the end of the first experimental phase.

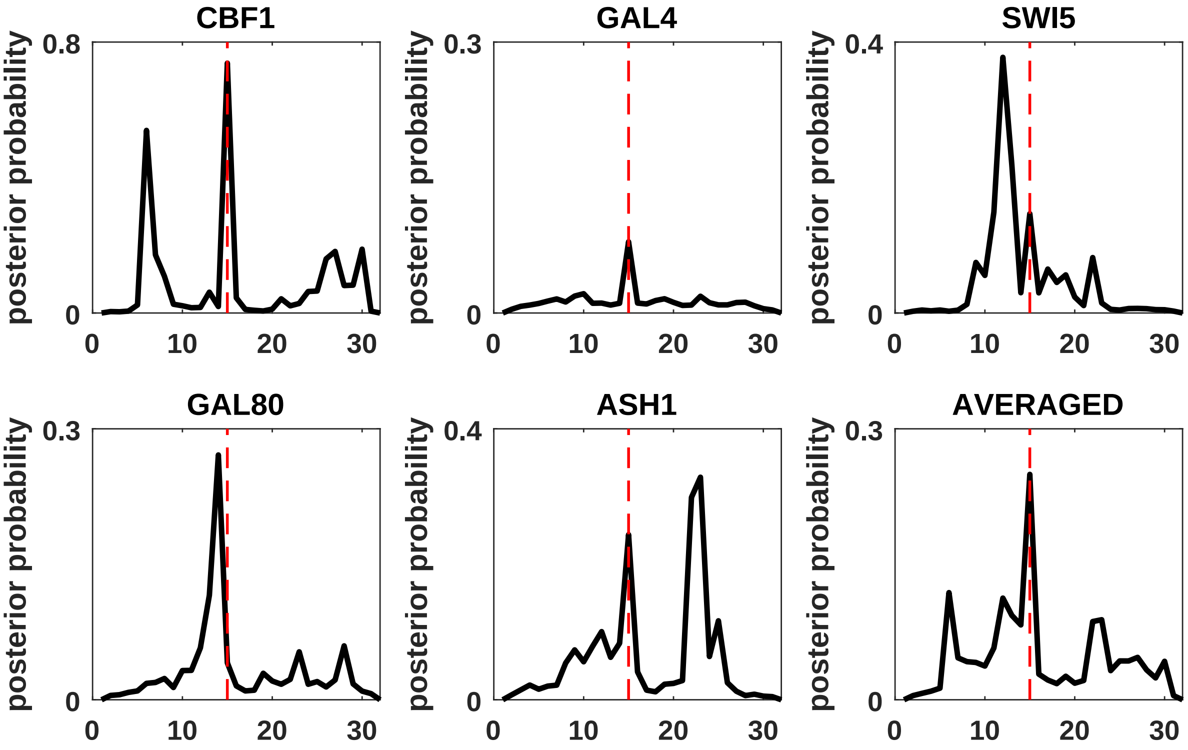

Figure 6: **Inferred changepoint locations for the yeast data.** For each gene there is a panel showing the average posterior probability of a changepoint (horizontal axis) at a specific time point (vertical axis). The vertical dotted lines indicate the true transition from galactose to glucose metabolism. The bottom right panel shows the average posterior probabilities, averaged across the 5 individual genes.

G.3 - Concrete network predictions (supplement for Section 5.2)

In this subsection of the supplementary material we show concrete network structure predictions that can be obtained from the yeast data.

For the EWC NH-DBN and for the two limiting models, namely the uncoupled (M2) and the coupled (M3) NH-DBN, we also averaged the model-specific edge scores for the yeast data across the $H = 10$ independent RJMCMC simulations. Figure 7 shows the resulting model-specific precision-recall curves, based on average edge scores.

For each of the three NH-DBNs we then extracted a network prediction, showing the $M = 8$ edges with the highest scores. Figure 8 shows the true network and the three inferred networks. The new model infers the highest scores for 6 true edges, while the competing NH-DBNs have already a false positive among the three highest-scoring edges, leading to reduced AUC values. The predictions of the uncoupled and the edge-wise coupled NH-DBN are similar. But the uncoupled NH-DBN assigns its third highest score to the false edge $GAL80 \rightarrow ASH1$, while the proposed model assigns its six highest scores to true edges. The fully coupled NH-DBN infers two different false positive edges, and the edge $GAL4 \rightarrow CBF1$ gets the third highest score.

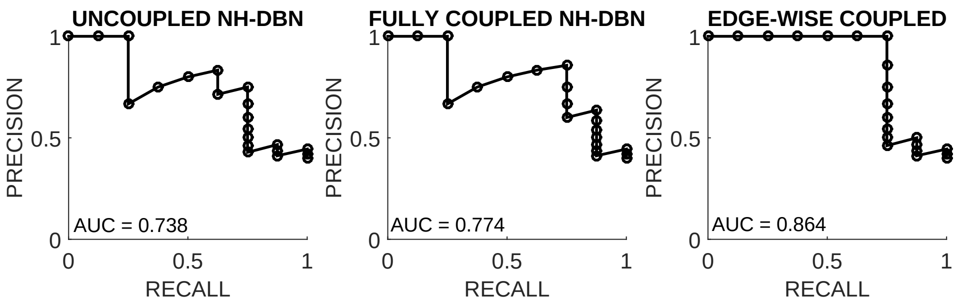

Figure 7: **Precision-recall curves for yeast network.** For the uncoupled (M2), the fully coupled (M3) and the new EWC NH-DBN, we computed the average edge scores across the $H = 10$ simulations. The figure shows the resulting model-specific precision recall curves. In each panel, the first point $(0, 1)$ is a pseudo point (the starting point). Unlike Figures 6-7 of the main paper, which show average AUC values, this figure gives the AUC values of the average edge scores.

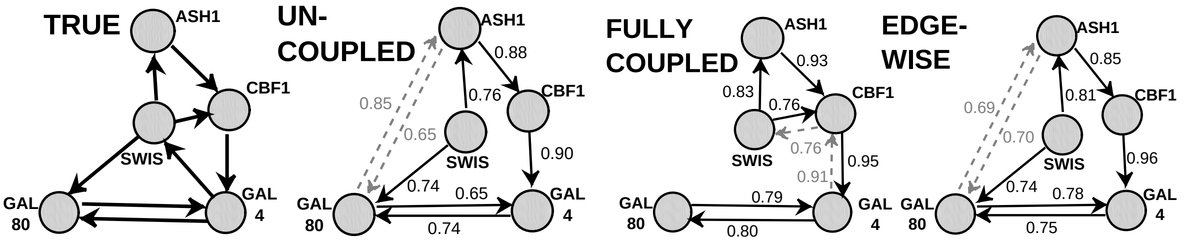

Figure 8: **The true and the three predicted yeast networks.** We averaged the model-specific edge scores across $H = 10$ independent RJMCMC simulations. As the true network has $M = 8$ edges, we extracted for each model the 8 edges with the highest scores. Grey edges refer to false positive edges. The edge labels give the edge scores. All three predicted networks yield a 'recall' of 75% and a 'precision' of 75%; i.e. they refer to the points $(0.75, 0.75)$ in the precision-recall curves in Figure 7.

Part H - Changepoint inference for Arabidopsis data (see Section 5.3 of the main paper)

Figures 9-10 show the inferred marginal posterior probability of the changepoint locations for the Arabidopsis data. Figure 9 shows the posterior probabilities for the $N = 9$ individual genes, while Figure 10 shows the average posterior probability (averaged across the individual genes). In this application, the three changepoints refer to transitions among four experiments. As described in more detail in Part C of this supplementary material, data from four individual experiments (E1-E4) were merged into one single time series, and the three changepoints here refer to the three boundaries between the experiments.
Figure 9 shows that the changepoints (transitions) yield rather low peaks for most of the genes and that some genes (ELF4, PRR9 and PRR5) seem to stay unaffected from the changing experimental conditions. A possible explanation is that the most important factor (the artificially induced dark:light phase) was the same for all experiments (constant light condition). Only the dark:light phases, in which the plants were entrained, differed between the experiments. For the two central regulator genes of the circadian clock network (LHY and TOC1), the posterior probability peaks around the middle changepoint. The same applies to the genes CCA1, ELF3 and PRR3, while the posterior probability for gene GI has a strong peak at the first boundary (between the first two experiments). The averaged posterior probability in Figure 10 has peaks around the first two boundaries, while the third boundary stays undetected.

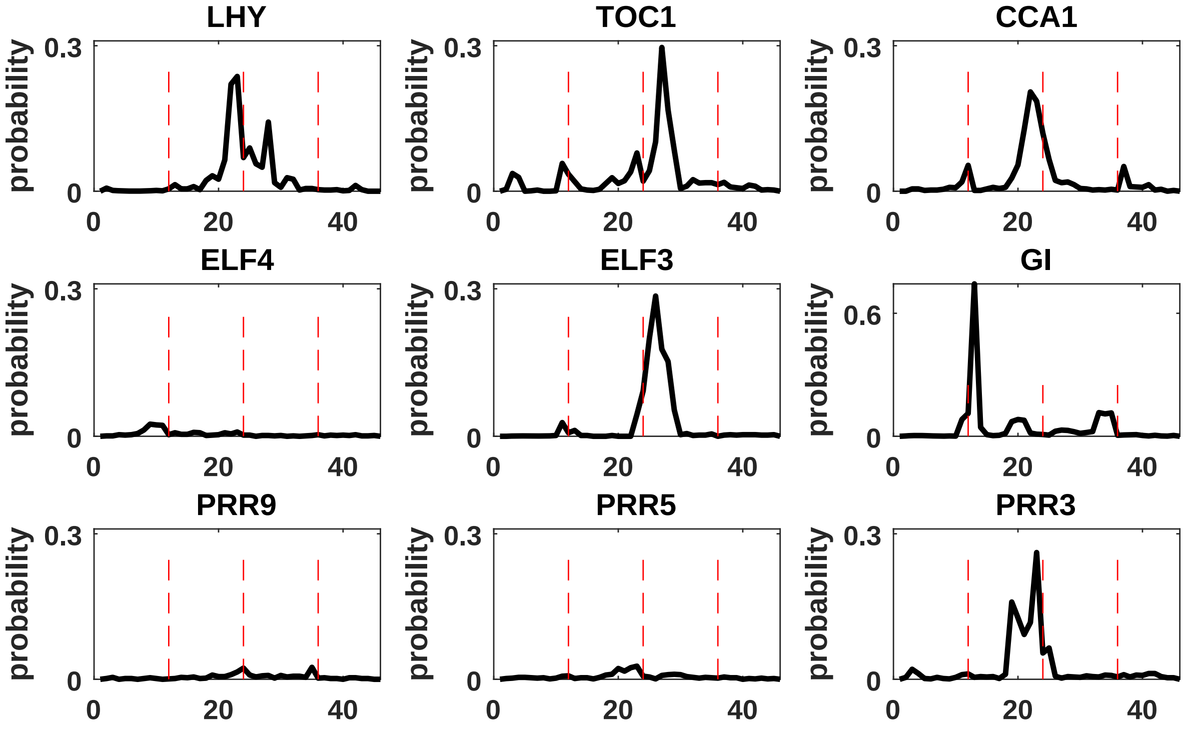

Figure 9: **Inferred changepoint locations for the Arabidopsis data - Part 1/2.** For each gene there is a panel showing the posterior probability of a changepoint (horizontal axis) at a specific time point (vertical axis). The vertical dotted lines indicate the transitions between the four individual time series. The average posterior probability curve, averaged across the individual genes, is shown in Figure 10.

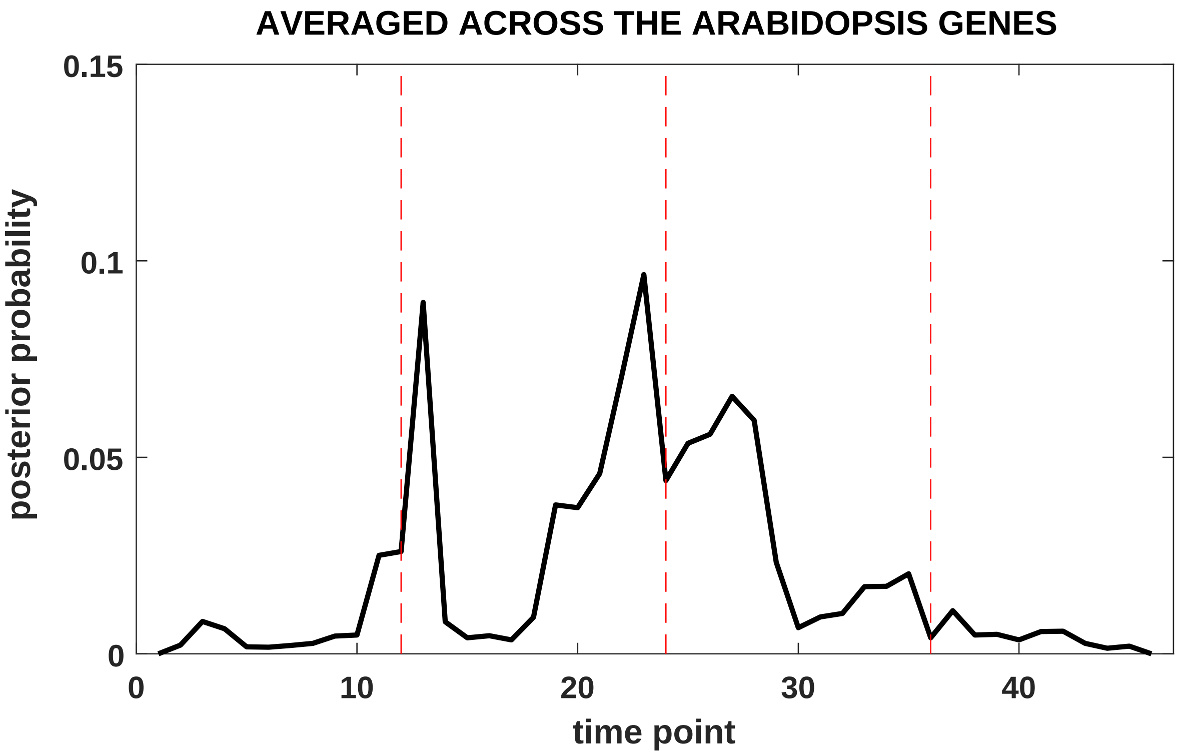

Figure 10: **Inferred changepoint locations for the Arabidopsis data - Part 2/2.** The figure shows the averaged posterior probability of a changepoint (horizontal axis) at a specific time point (vertical axis). It has been averaged across the posterior probabilities of the $N = 9$ individual genes, shown in Figure 9.

Part I - Non-linear vs. non-homogeneous models (see Section 1 of the main paper)

I.1 - Conceptual illustration and toy example

In this section of the supplementary material we illustrate the conceptual difference between non-linear and non-homogeneous network reconstruction methods. In modern computational biology, researchers often choose one of the two modelling paradigms when developing new network reconstruction methods.
We start with an illustrative example: Figure 13 shows four possible types of regulatory relationships between a regulator gene and a regulatee gene. The conventional dynamic Bayesian network (DBN) models, like the M1 model from Section 2.6 of the main paper, assume the relationship to be homogeneous and linear (see top left panel of Figure 13). But when dealing with gene regulatory interactions, neither the linearity nor the homogeneity assumption is realistic. Therefore more advanced network reconstruction models have been proposed. For example, the models M2-M7 (Section 2.6 of the main paper), A3-A4 (Section 2.7 of the main paper) and the newly proposed EWC NH-DBN stay with linear models, but relax the homogeneity assumption by segmenting the data. That is, they aim to infer time-varying linear relationships between a regulator and its regulatee (see bottom left panel of Figure 13). Other models, like the models A1, A6, A7, and A8 (Section 2.7 of the main paper) stay with the homogeneity assumption, but relax the linearity assumption. They aim to infer homogeneous non-linear relationships between a regulator and its regulatee (see top right panel of Figure 13). One could obviously also combine both paradigms (see bottom right panel of Figure 13). However, to the best of our knowledge, no non-homogeneous non-linear model has been proposed in the literature yet. Although the model turned out not to yield convincing network reconstruction results, the A2 model (NH-DBN with covariate transformations) from Section 2.7 of the main paper is effectively a non-homogeneous non-linear model.

To show empirically that homogeneous non-linear models cannot be used for inferring non-homogeneous linear relationships, we now consider a synthetic data example, see Figure 11. In the example the autoregressive variable X is a regulator of the variable Y and the regulatory effect is subject to a time lag, i.e. $X(t)$ is a covariate for $Y(t+1)$. The detailed mathematical relationships are provided in the bottom left box of Figure 11. When plotting the temporal profiles (see top left panel), the underlying relationship can hardly be spotted. The scatter plot '$Y(t+1)$ against $X(t)$' in the top right panel does not reveal a functional relationship either. The Pearson correlation coefficient, as a measure for the strength of the linear relationship, is equal to $\rho \approx 0.01$. Also the cubic polynomial, which we fitted to the data, does not reveal a functional relationship (no significant regression coefficients). However, when dividing the data into two segments ($t \leq 25$ and $t > 25$) and computing the segment-specific Pearson correlation coefficients, we obtain $\rho_1 \approx -0.53$ and $\rho_2 \approx 0.61$. This indicates that there are segment-specific linear relationships. We therefore fitted segment-specific linear models to the data and found that intercept and slope of both lines were significant. Hence, we conclude that a homogeneous non-linear model is not appropriate for inferring a non-homogeneous linear relationship. In the next subsection (H.2) we have a brief look at the yeast data (see Section 4.2 of the main paper).

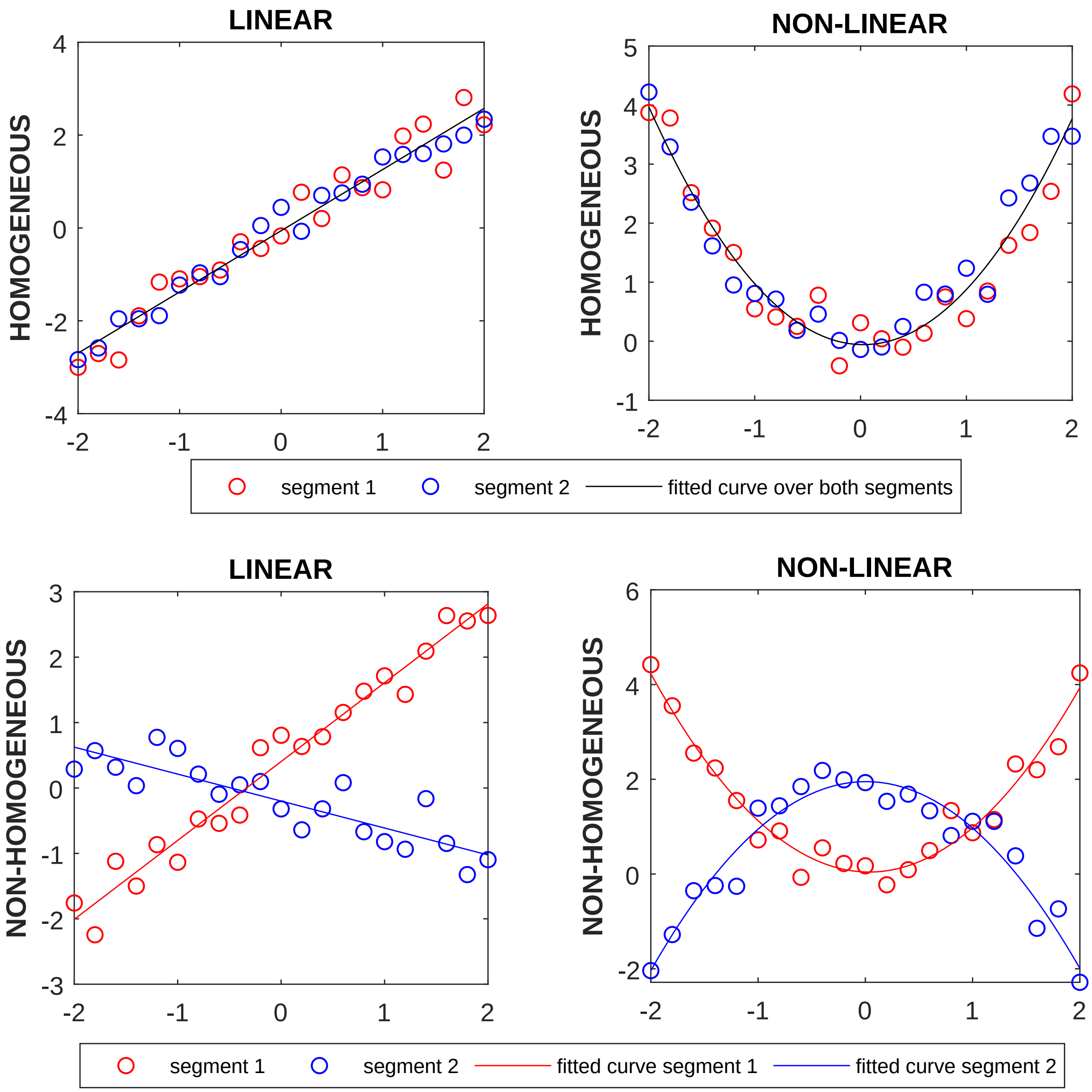

Figure 11: **Illustration of the conceptual differences between four types of regulatory relationships.** The figure has four panels, and each panel shows a scatter plot of the values of a regulator (horizontal axis) and a regulatee (vertical axis). When the data points have been measured over time, they can be separated into two parts: segment 1 (red data points) and segment 2 (blue data points). The two rows refer to homogeneous (top) and non-homogeneous (bottom) relationships; the two columns refer to linear (left) and non-linear (right) regulatory relationships. For illustrative purposes, the solid curves show the true relationships between the two variables. The data points were obtained by adding Gaussian distributed noise. For homogeneous relationships the curve is the same for both segments (black curves), for non-homogeneous relationships the curve is segment-specific (red and blue curve). Linear relationships can be described by lines, and for non-linear relationships we decided to use quadratic functions, though we could also have used another type of non-linear function.

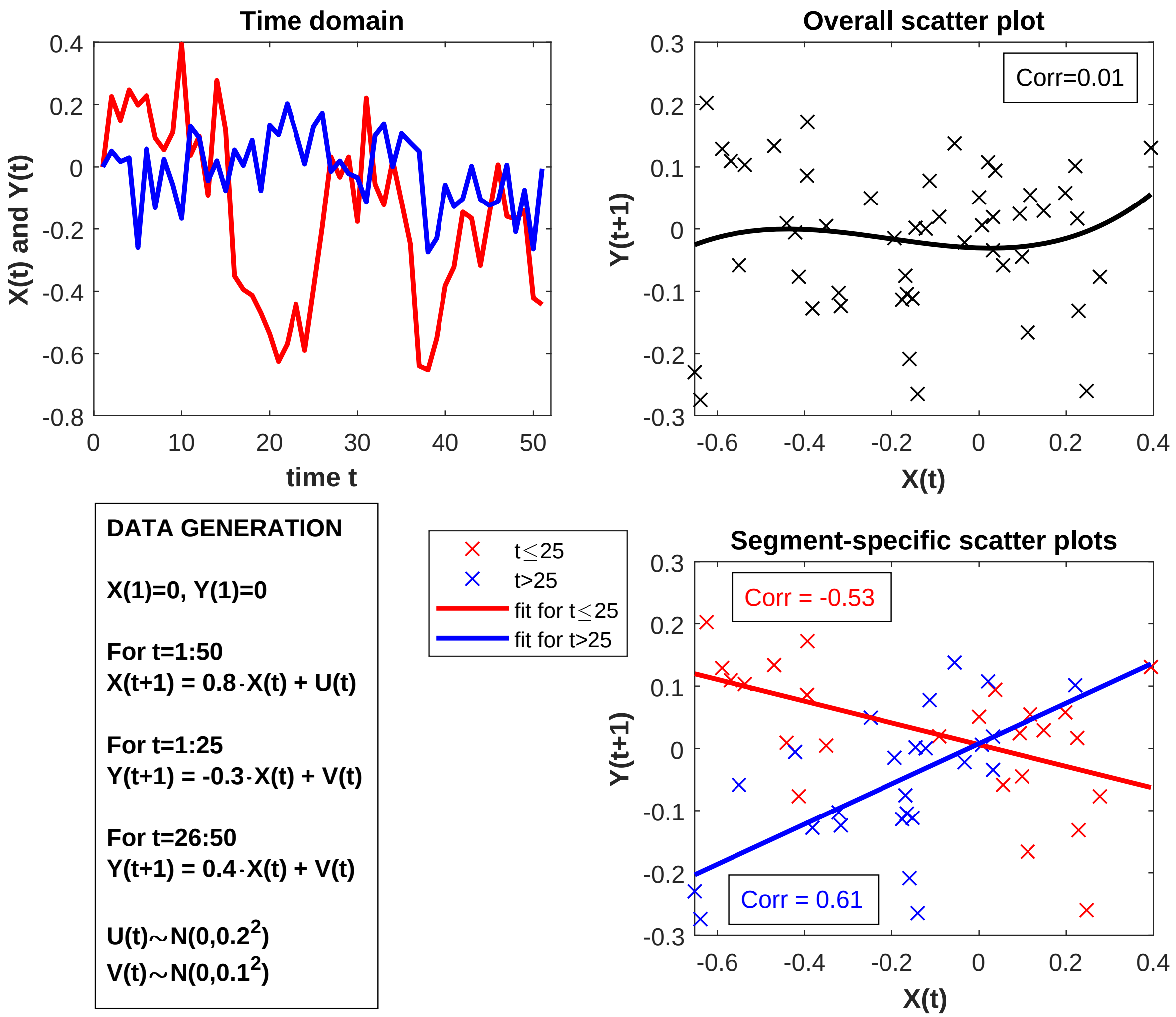

Figure 12: **Toy data example.** To demonstrate that homogeneous non-linear models are inappropriate for learning non-homogeneous linear relationships, we consider a small synthetic data example. For two variables X and Y we generate a data instantiation $(X(t), Y(t))_{t=1,\ldots,50}$, as described in the bottom left box. The top left panel shows the sampled trajectories of X and Y. The top right panel shows a scatter plot, where $Y(t+1)$ (vertical axis) has been plotted against $X(t)$ (horizontal axis). The Pearson correlation is 0.01. In addition a cubic polynomial has been fitted to the data (black solid line). In the scatter plot in the bottom right panel the data points have been divided into two segments: $t \leq 25$ (red) and $t > 25$ (blue), and to both segments a linear model has been fitted. The segment-specific Pearson correlations are -0.53 and 0.61. See main text for further details.

I.2 - The yeast data

We now have a brief look at the yeast data, so as to get a first impression whether a homogeneous non-linear or a non-homogeneous linear model might be more appropriate for inferring the yeast network structure. Figure 13 shows scatter plots of all possible regulatory relationships in the yeast network, and almost none of the scatter plots indicates a (clear) functional relationship. We would thus conclude from Figure 13 that a homogeneous (linear or non-linear) model might not be optimal. We note that it cannot be seen from Figure 13 whether the relationships are non-homogeneous (segment-specific). To search for non-homogeneous functional relationships, the data have to be segmented and the segment-specific scatter plots have to be considered. Figure 14 shows the same scatter plots like Figure 13, but the data points from the two segments (galactose and glucose metabolism) are now coloured differently (red and blue). Moreover, we fitted segment-specific linear models to the data (red and blue solid lines). Although potentially only a rather subjective impression, we would argue that some of the scatter plots suggest that there might be segment-specific linear relationships between the genes. In particular, we note that clearer trends might be found when inferring the best segmentations.

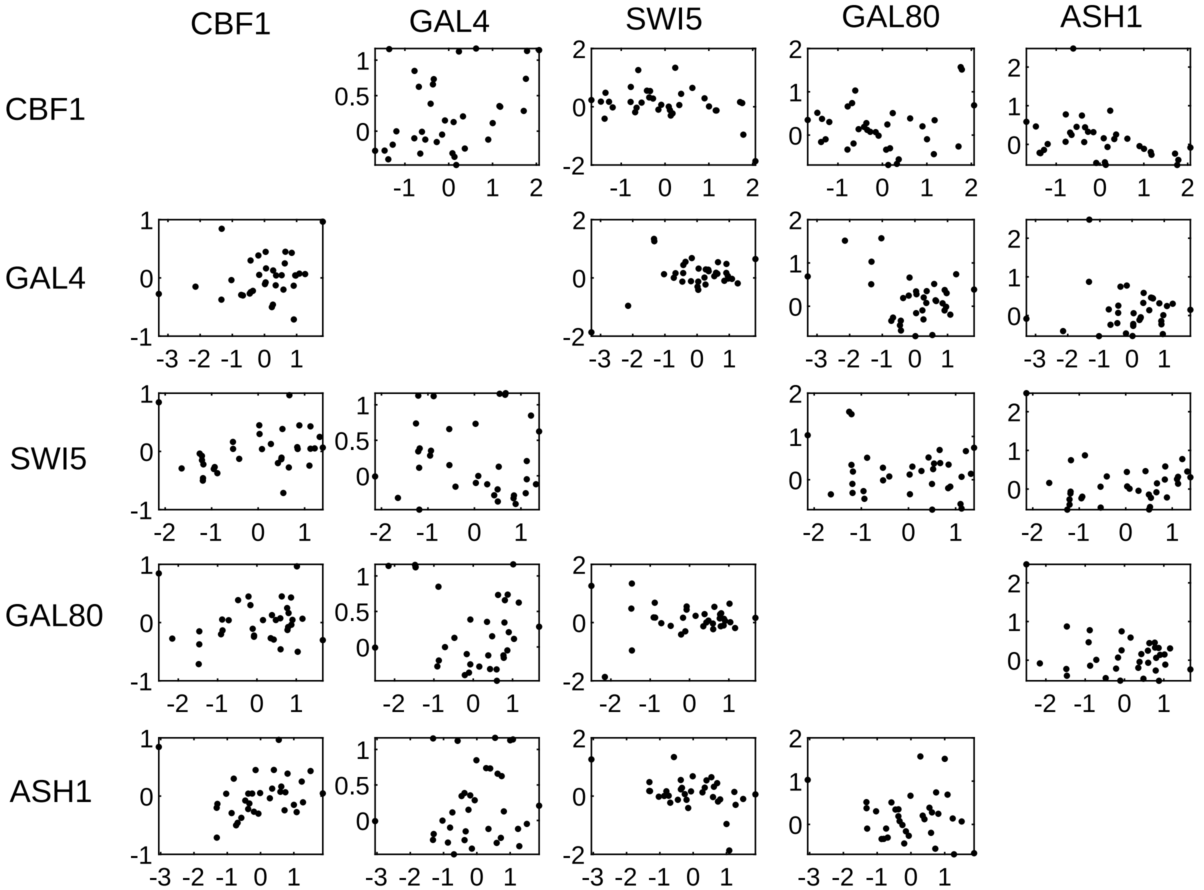

Figure 13: **Scatter plots of all possible regulatory interactions in the yeast network.** In each panel the values of one potential regulatee gene at time points $t = 2, \ldots, 33$ (vertical axis) have been plotted against the values of another potential regulator gene at time points $t = 1, \ldots, 32$ (horizontal axis). The rows refer to the regulators; the columns refer to the regulatees.

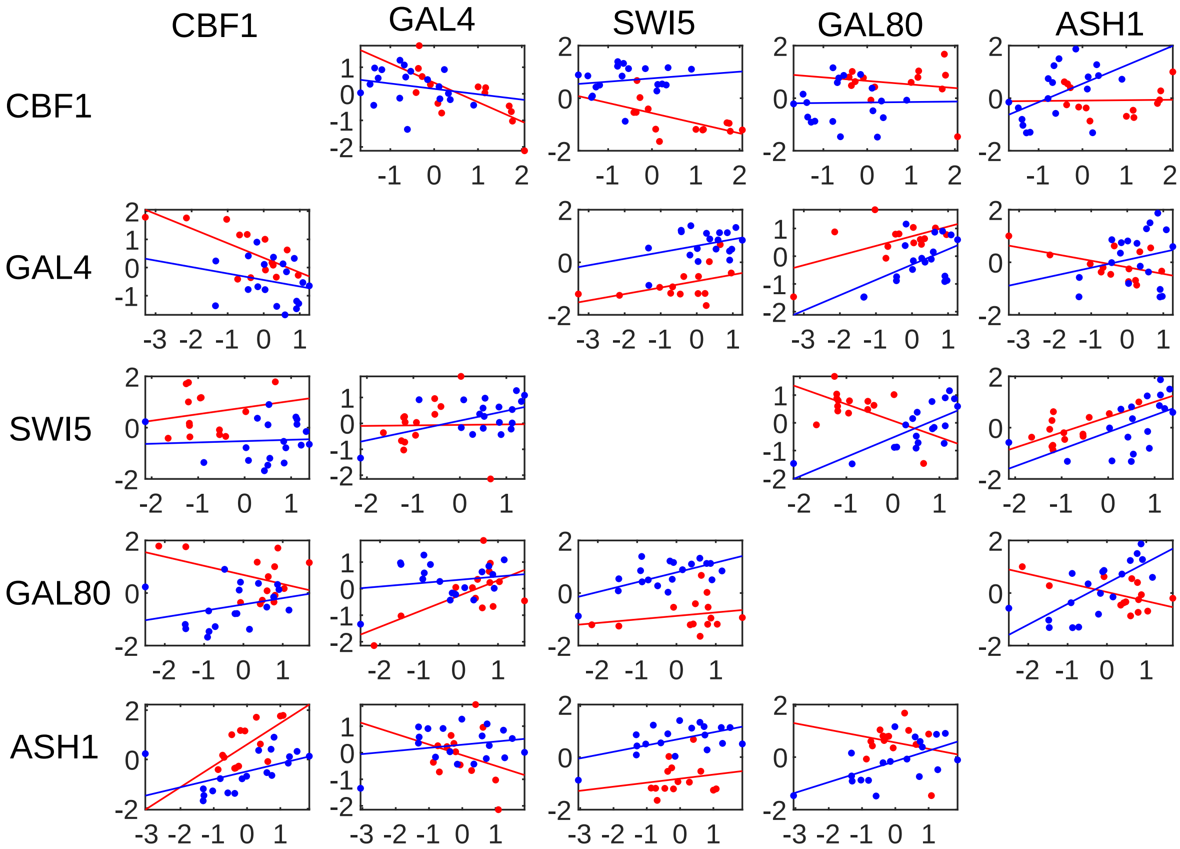

Figure 14: **Overlaid segment-specific scatter plots of all possible regulatory interactions in the yeast network.** Like in Figure 13, in each panel the values of one potential regulatee gene (vertical axis) have been plotted against the values of another potential regulator gene (horizontal axis). Rows refer to the regulators; columns refer to the regulatees. But unlike in Figure 13, it is now distinguished between both segments: data points measured under galactose metabolism are in red, and data points measured under glucose metabolism are in blue. In addition, segment-specific linear models have been fitted to the data (red and blue solid lines). For example, the edge ASH1→ CBF1 is a true edge of the yeast network, and the relationship between ASH1 (at t) and CBF1 (at $t+1$) in the bottom left panel could be of a non-homogeneous linear nature.

References

Bishop, C. M. (2006) *Pattern Recognition and Machine Learning.* Springer, Singapore.

Brooks, S. and Gelman, A. (1998) General methods for monitoring convergence of iterative simulations. *Journal of Computational and Graphial Statistics*, **7**, 434–455.

Cantone, I., Marucci, L., Iorio, F., Ricci, M., Belcastro, V., Bansal, M., Santini, S., di Bernardo, M., di Bernardo, D. and Cosma, M. (2009) A yeast synthetic network for in vivo assessment of reverse-engineering and modeling approaches. *Cell*, **137**, 172–181.

Edwards, K., Anderson, P., Hall, A., Salathia, N., Locke, J., Lynn, J., Straume, M., Smith, J. and Millar, A. (2006) Flowering locus C mediates natural variation in the high-temperature response of the Arabidopsis circadian clock. *The Plant Cell*, **18**, 639–650.

Grzegorczyk, M., Husmeier, D., Edwards, K., Ghazal, P. and Millar, A. (2008) Modelling non-stationary gene regulatory processes with a non-homogeneous Bayesian network and the allocation sampler. *Bioinformatics*, **24**, 2071–2078.

Mockler, T. C., Michael, T. P., Priest, H. D., Shen, R., Sullivan, C. M., Givan, S. A., McEntee, C., Kay, S. A. and Chory, J. (2007) The diurnal project: Diurnal and circadian expression profiling, model-based pattern matching and promoter analysis. *Cold Spring Harbor Symposia on Quantitative Biology*, **72**, 353–363.
